# p53 wild-type/low c-Myc-expressing ONS76 pediatric medulloblastoma cells become senescent after gamma-irradiation and can be eliminated by senolytic substances

**DOI:** 10.1038/s41420-026-03404-4

**Published:** 2026-10-08

**Authors:** Jessica Sonntag, Ronja-Linda Preugschas, Danny Jazmati, Nan Qin, Judith Neuwahl, Christiane Matuschek, Marc Remke, Wilfried Budach, Juliane Hörner-Rieber, Dennis Sohn

**Affiliations:** 1 Laboratory of Molecular Radiooncology, Clinic and Policlinic for Radiation Therapy and Radiooncology, Medical Faculty and University Hospital Düsseldorf, Heinrich-Heine-University Düsseldorf, Düsseldorf, Germany; 2 Clinic and Policlinic for Radiation Therapy and Radiooncology, Medical Faculty and University Hospital Düsseldorf, Heinrich-Heine-University Düsseldorf, Düsseldorf, Germany; 3 Department of Hematology, Oncology and Clinical Immunology, Medical Faculty and University Hospital Düsseldorf, Heinrich-Heine-University Düsseldorf, Düsseldorf, Germany; 4 Department of Radiation Oncology, Medical School and University, Medical Center OWL, Klinikum Mitte, Bielefeld University, Bielefeld, Germany; 5 Department of Pediatric Oncology and Hematology, Saarland University, Medical Center, Homburg, Germany

**Keywords:** CNS cancer, Radiotherapy, Cell death

## Abstract

Craniospinal irradiation (CSI) is an indispensable part of oncological treatment for pediatric medulloblastoma. However, despite significantly improving oncological outcome, it is also associated with severe short- and long-term side effects such as inflammation and impaired neurocognition. The presence of senescent cells has been identified as a potential contributing factor to these issues. In our study, we demonstrate that p53 wild-type and c-Myc-low-expressing ONS76 medulloblastoma cells underwent senescence, a terminal and stable cell cycle arrest, after exposure to gamma irradiation (γIR). In contrast, p53-mutated UW228-3 cells exhibited apoptosis following the same treatment. ONS76 cells displayed typical senescence properties, including cell and nuclei enlargement, an active DNA damage response with stabilization of p53 and increased expression of its target gene, the CDK inhibitor p21^WAF1/CIP1^, as well as elevated senescence-associated beta-galactosidase activity and lipofuscin accumulation. Knockdown of p53 or p21 expression with siRNAs abolished γIR-mediated senescence. Furthermore, overexpression of c-Myc resulted in a partial shift from senescence to cell death induction after γIR treatment, which was inhibited by using the caspase-inhibitor q-VD-OPh. The γIR-exposed ONS76 cells showed susceptibility to senolytic substances, such as the BCL-2 family inhibitor Navitoclax and the PI3K-inhibitors PX-866 and BAY80-6946, which specifically target senescent cells. In conclusion, employing senolytics on irradiated medulloblastoma cells with wild-type p53 and low c-Myc expression may lead to reduced therapeutic side effects as well as attenuate tumor relapse and potentially minimize CSI doses required for effective tumor cell elimination.

## Introduction

Medulloblastoma is the most common malignant brain tumor in children [1]. The WHO categorizes this entity into four groups based on genetic and histological characteristics: (I) WNT-activated, (II) SHH-activated with p53 wild-type, (III) SHH-activated with mutated p53, and (IV) non-WNT/Non-SHH activated (former “group 3” and “group 4”) [2, 3]. Recent research has expanded this classification by incorporating additional factors such as copy number alterations, DNA methylation, and gene expression data from 763 primary samples and identified 12 subtypes (WNT α−β, SHH α−δ, and Group 3 and 4 α−γ) [4]. These exhibit distinct features in terms of genome alterations, occurrence and disease manifestation, as well as treatment options.

The standard therapeutic regimen for pediatric medulloblastoma typically comprises maximal safe resection, followed by craniospinal irradiation (CSI) and chemotherapy [5]. However, CSI increases the occurrence of endocrine dysfunction and neurocognitive impairments [6–8]. Given the vulnerability of immature neural tissue, the potential risks must be carefully balanced against the anticipated benefits. Therefore, a personalized therapeutic approach would be desirable. A recent study omitting the CSI in WNT-medulloblastoma had to be terminated prematurely because all patients rapidly relapsed [9], indicating the importance of radiotherapy for treating WNT-medulloblastoma [10] and most probably also other subgroups.

At the cellular level, radiotherapy causes massive DNA damage, which ultimately results in the induction of irreversible cell fates [11]. The tumor suppressor p53 orchestrates these processes depending on several factors such as cell type and damage [12]. After irreparable DNA damage, p53, as a central transcription factor, induces opposing cell fates such as apoptosis or senescence, a terminal and stable cell cycle arrest [13]. These decisions are executed by p53 target genes, such as the apoptosis-inducing Bcl-2-family members PMAIP1 and BBC3 (encoding for Noxa and Puma) or the essential senescence-mediating CDK-inhibitor CDKN1A (p21^WAF1/CIP1^) [14]. In the absence of p53, cells undergo a complex process called mitotic catastrophe that is instigated by unrepairable DNA damage preventing correct mitotic progression. This often results in cell death by a very complex phenotype and is hypothesized to be the main cause of therapy-induced cell death in p53-mutated or -deleted tumor cells [15, 16].

Cellular senescence was initially identified as a critical tumor suppressor mechanism [17]. Replicative senescence controls the cellular division capacity via progressive shortening of telomere length during each round of genome replication. Although this process heavily contributes to organismal aging, it effectively delays tumorigenesis, as cells must first overcome this proliferation block. In addition, premature oncogene-induced senescence directly inhibits tumor cell transformation, e.g., after activation of proto-oncogenes such as c-Myc or Ras. This results in the activation of the ARF signaling pathway that blocks proliferation and manifests senescence together with another CDK-inhibitor, p16^INK4A^, and p53/p21 [18, 19].

Until recently, the importance of cellular senescence has been neglected in the oncological clinical setting. However, senescent cells persist in high amounts during aging or after DNA damage-based anti-cancer therapy and develop a so-called senescence-associated secretory phenotype (SASP). It consists of a plethora of different cytokines, chemokines, and other secreted factors, which among other consequences, lead to tissue microinflammation [17, 20]. Thereby, senescent cells exert a huge impact on their surrounding cells. Their timely generation is important for physiological wound healing [21, 22]; their chronic persistence, however, is detrimental for tissue function and supports tumor relapse [23]. Therefore, novel so-called senolytic substances are developed to specifically target and eliminate death-resistant senescent cells. In addition to the already demonstrated positive impact of removing senescent cells to counter aging-related pathologies [24, 25], it is proposed that such agents should also inhibit the in vivo occurrence of short- and long-term side effects of chemo- and radiotherapy [26].

Several observations suggest a clinically relevant connection between medulloblastoma and cellular senescence. Firstly, oncogene-induced senescence has been described to be an especially important process in inhibiting medulloblastoma progression [27]. In a Ptch1 + /- model for pre-neoplastic medulloblastoma lesions, the full loss of Ptch1 did not result in medulloblastoma formation but instead in massive cellular senescence [28]. Only cells with spontaneous mutation of either p53 or p16 were able to escape senescence and form a fully developed medulloblastoma. This demonstrates that cellular senescence represents a default tumor suppressor program in such precursor cells. Furthermore, CSI therapy is essential for curing medulloblastoma but also responsible for long-term negative side effects, resulting in accelerated brain aging that is especially detrimental in young surviving patients [29].

However, the induction of senescence during CSI has not been analyzed thoroughly before. Therefore, we exposed two SHH-activated medulloblastoma cell lines, ONS76 (p53 wild-type) and UW228-3 (p53-mutated), to different doses of gamma-irradiation (γIR) and determined their cellular responses. In addition, we employed the same cell lines overexpressing c-Myc to analyze if this oncogene alters γIR-induced cell fates.

## Results

### γIR induces a senescent-like morphology and SA-beta-galactosidase activity in wild-type p53 ONS76 cells

Whereas cell types such as lymphocytes predominantly undergo apoptosis after suffering from massive DNA damage, fibroblasts or epithelial cells usually react by becoming senescent [30]. Because radiotherapy is both a potent activator of p53/p21-dependent senescence and an important component of pediatric medulloblastoma treatment, we analyzed the γIR-response of ONS76 cells expressing functional wild-type p53. As a counterpart, we employed dysfunctional p53-mutated UW228-3 cells that, similarly to ONS76, are characterized by SHH activation [31].

**Fig. 1:**
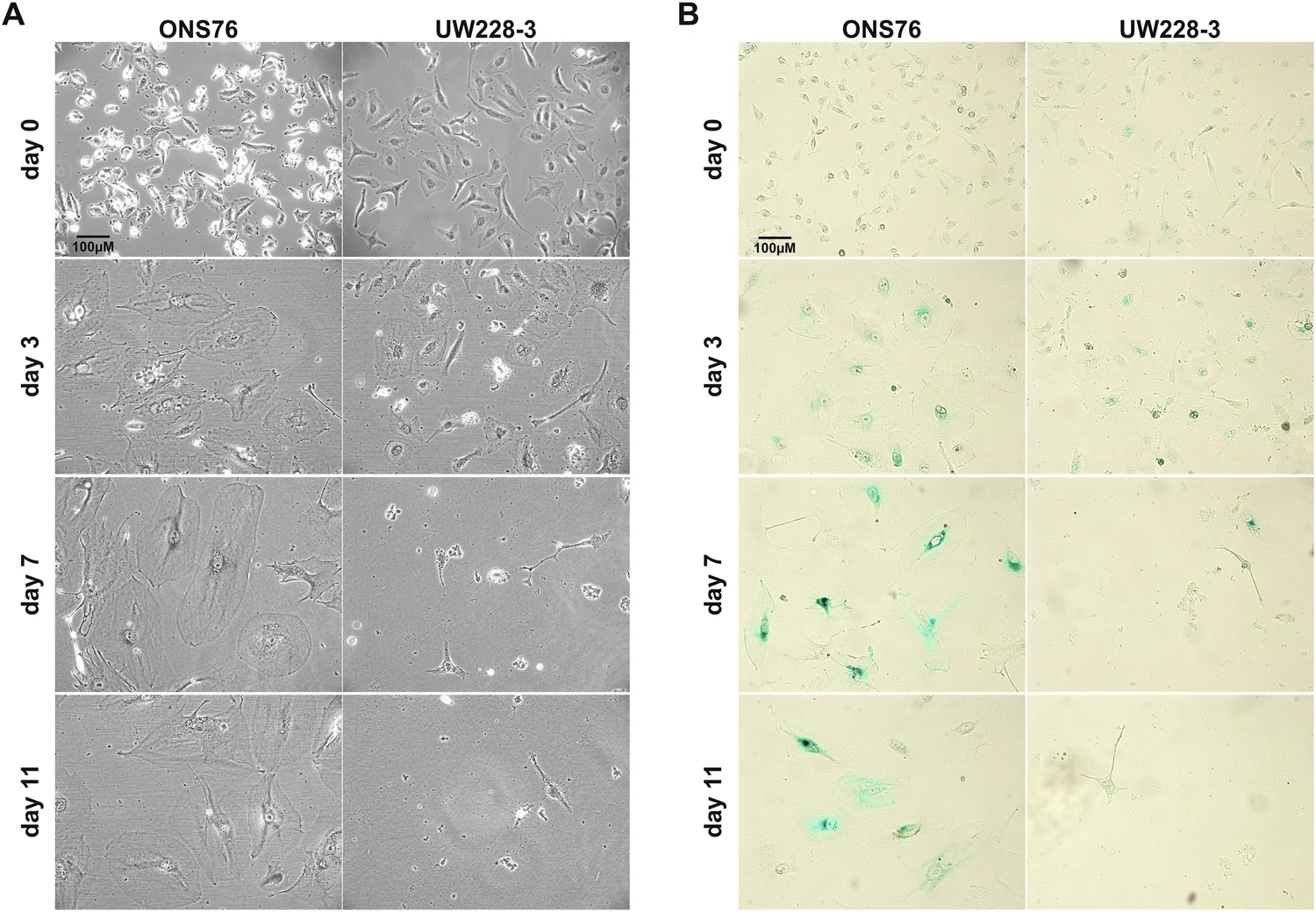
Irradiated ONS76 cells are positive for senescence-associated beta-galactosidase activity and display a senescent morphology. The two pediatric medulloblastoma cell lines ONS76 (p53 wild-type) and UW228-3 (p53-mut.) were exposed to 20 Gy γIR. After the indicated time periods, cells were stained for senescence-associated beta-galactosidase activity (manifested in blue) and documented microscopically in phase contrast (**A**) and brightfield (**B**) using a Zeiss Axio Vert.A1 microscope. With increasing incubation times after irradiation, ONS76 display a strong blue staining and morphological changes, such as an increase in cell and nucleus size, reminiscing of cellular senescence, whereas UW228-3 cells detach from the bottom and die. All pictures were taken with the same 10× objective and the included 100 µm scale bar is representative for all micrographs. The pictures chosen are representative for at least three independent biological experiments. Please note that detached and dead cells are aspirated during the SA-beta-Galactosidase staining procedure and are therefore not present anymore, in contrast to Supplementary Fig. 1, in which the cells were photographed under culturing conditions.

In proliferation-based radioresistance assays, both cell lines display similar calculated SF2-values (surviving fraction 2 Gy) [32]. However, upon microscopic inspection, their γIR response following a 20 Gy dose was fundamentally different. UW228-3 cells detached and degraded completely after 3–7 days, whereas ONS76 cells remained attached and exhibited altered morphology for more than 50 days (Fig. 1; Supplementary Fig. 1A; data not shown). Their appearance became very flat with an enormous increase in cell and nucleus size, although the nuclei appear intact (Fig. 1A; Supplementary Fig. 1A). In addition, ONS76 cells developed increased senescence-associated beta-galactosidase (SA-βG) activity over the course of 11 days (Fig. 1B). Such observations are typical for a senescent phenotype [33]. The p53-mutated cell line UW228-3, however, only showed a slightly elevated SA-βG activity at day 3 after γIR (Fig. 1B) without any typical morphological senescent features, and only a few still adherent viable cells remained visible from day 7 on (Fig. 1A; Supplementary Fig. 1A).

### γIR induces p53/p21-dependent senescence in ONS76 cells but apoptosis in p53-mutated UW228-3 cells

**Fig. 2:**
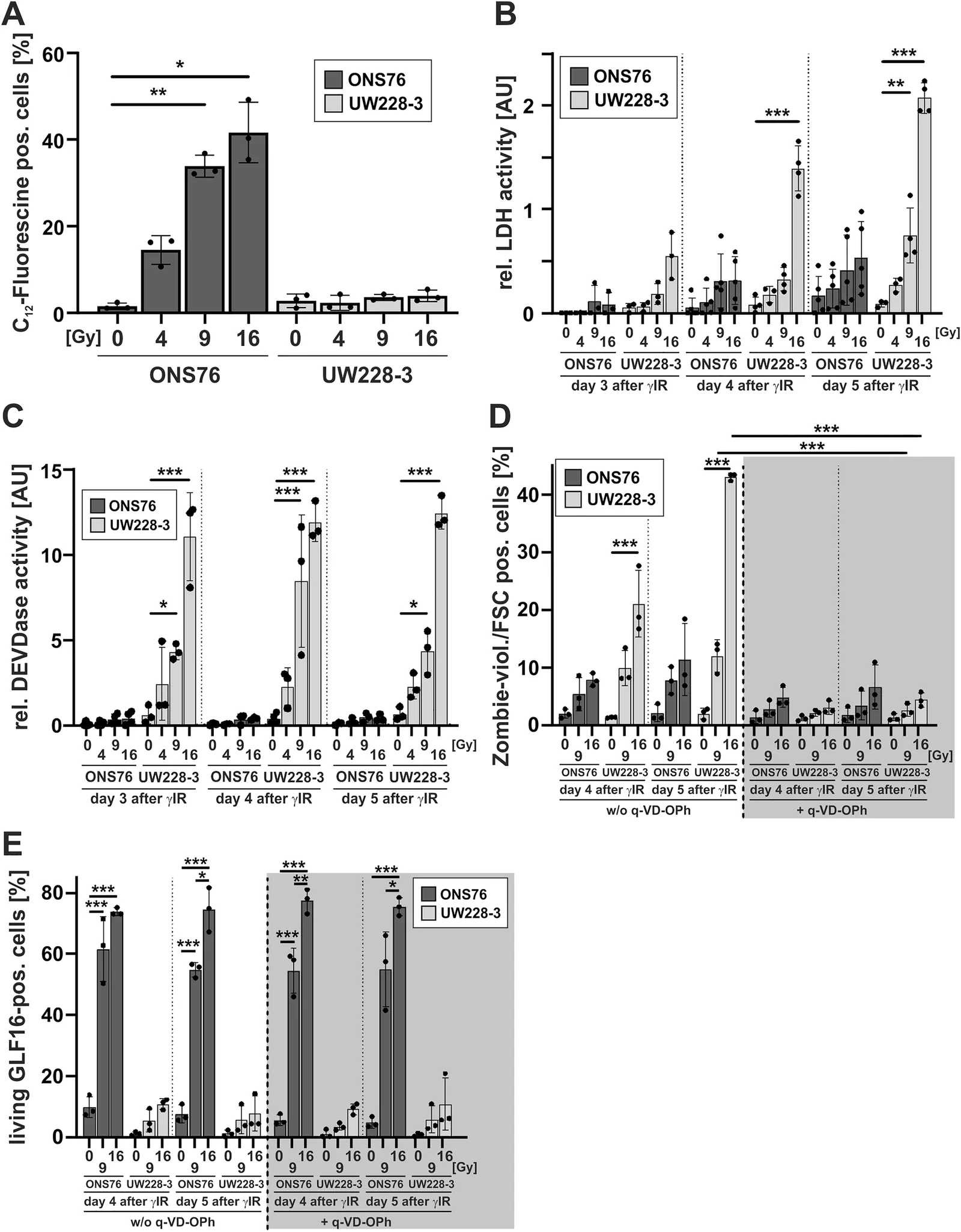
γIR induces apoptosis in p53-mutated UW228-3 cells, whereas senescence is initiated in p53 wild-type ONS76 cells. ONS76 and UW228-3 cells were exposed to the indicated doses γIR and harvested at day 5 (**A**) or at the indicated times (**B–E**). (**A**) SA-βG-positive cells, as indicated by a positive labeling with C_12_-fluorescine, were quantified flow cytometrically. Shown is the mean % of positive cells ± S.D. of three independent experiments. (**B**) Analysis of LDH activity in the supernatant of irradiated cells. Higher LDH activity resembles an increase in cell death. Shown are the mean activities ± S.D. of three to five independent experiments. (**C**) DEVDase-like activity in protein extracts of cells treated as described above. An increase in caspase activity indicates that the cell death detected in **B** is of an apoptotic nature. Shown are the mean activities ± S.D. of three to four independent experiments. (**D, E**) Flow cytometric analysis of cells stained positive with the amine-reactive dye Zombie-violet (**D**) or for lipofuscin-binding GLF16 (**E**) at days 4 and 5 after exposure to the indicated doses γIR in the absence (w/o q-VD-OPh) or presence (+10 µM q-VD-OPh, grey background) of a caspase-inhibitor. Shown is the mean % of positive cells ± S.D. of three independent experiments. Individual data points are displayed in the bar charts by small filled circles. *p < 0.05, **p < 0.01, ***p < 0.001.

To properly quantify our previous observations (Fig. 1), flow cytometric analysis using the SA-βG substrate C_12_-FDG was performed. Only within the ONS76 cell line a population with a substantial and dose-dependent increase in SA-beta-galactosidase activity was detected (Fig. 2A). No such cells were observed at day 5 after γIR treatment in the UW228-3 cell line. Instead, measurement of lactate dehydrogenase (LDH)-activity in the supernatant as a marker for plasma membrane destruction demonstrated that doses of 9 and 16 Gy resulted in significant cell death of UW228-3 cells (Fig. 2B). This was accompanied by an equal rise in DEVDase-like caspase-activity in cell extracts of similarly-treated UW228-3 (Fig. 2C). On the other hand, only marginal LDH- and no caspase-activities were detectable in irradiated ONS76 (Fig. 2B, C), demonstrating that only small background amounts of cell death occur.

As an additional, more reliable verification method, we employed the simultaneous flow cytometric detection of dead and senescent cells using Zombie-violet and the recently developed lipofuscin-binder GLF16 [34], respectively (Fig. 2D, E). In concordance with the obtained C_12_-FDG- (Fig. 2A) and LDH-data (Fig. 2B), but with a much better discrimination, we detected mainly senescent ONS76 cells after irradiation with 9 and 16 Gy (Fig. 2E), whereas UW228-3 demonstrated high amounts of cell death, especially after 16 Gy γIR (Fig. 2D). Addition of the pan-caspase-inhibitor q-VD-OPh (Fig. 2D, E; grey background) completely abolished γIR-mediated cell death, but senescence was not affected. Together with the elevated caspase activities (Fig. 2C), this indicates that γIR mainly induces apoptosis in UW228-3 cells.

**Fig. 3:**
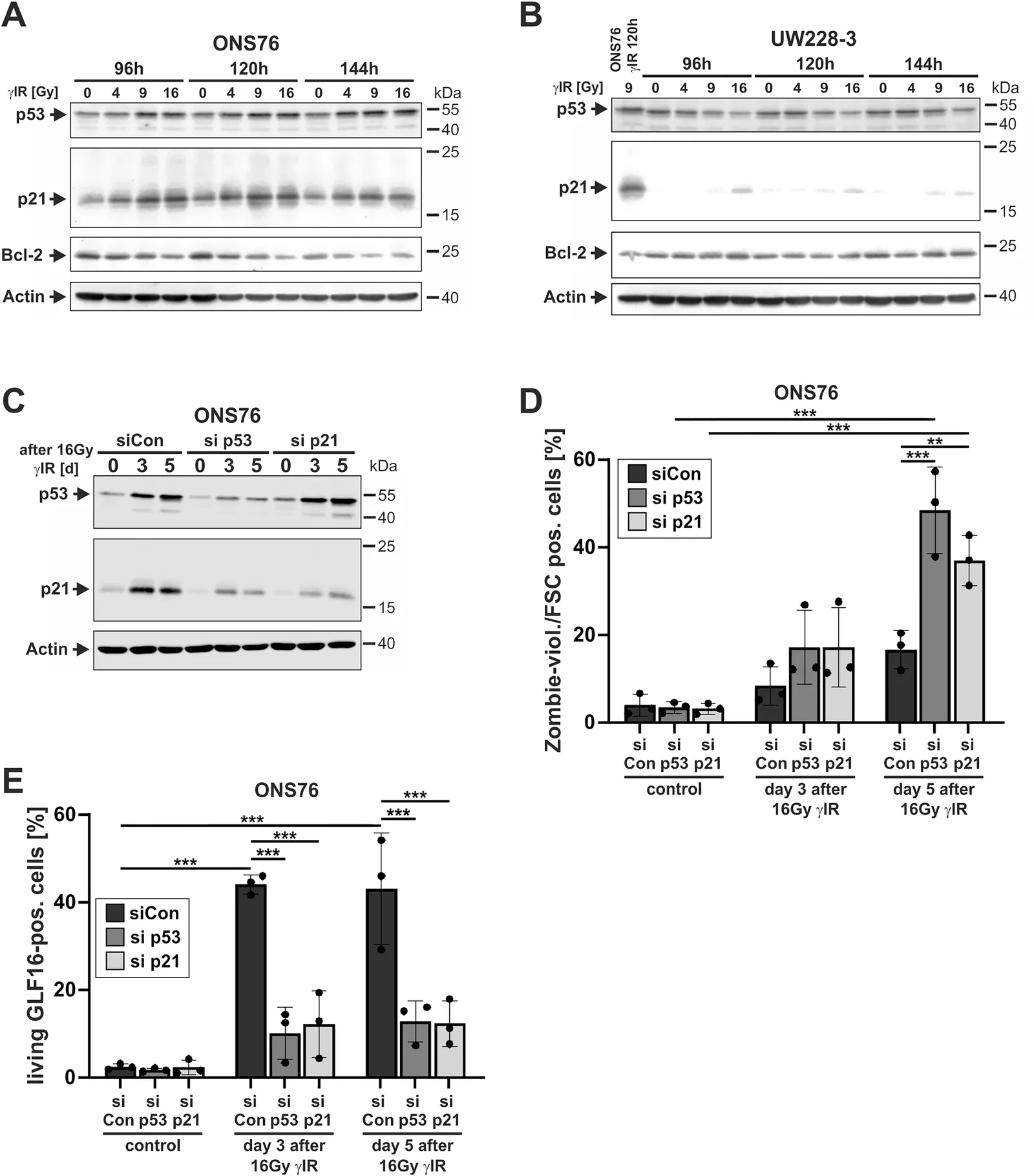
γIR induces p53/p21-dependent senescence in p53 wild-type ONS76 cells. ONS76 (**A**) and UW228-3 (**B**) cells were exposed to the indicated doses γIR and harvested at day 4, 5 and 6 after irradiation. Protein extracts were analyzed by western blotting for the expression of p53, p21, and Bcl-2, as well as actin as a housekeeper control. Please note that the ONS76 sample at day 5 after 9 Gy γIR was included on the UW228-3 blot (**B**) for a better comparison of protein expression levels between these two cell lines. This demonstrates the much lower p21 protein expression in irradiated p53-mutated UW228 cells when compared to irradiated p53 wild-type ONS76 cells. (**C–E**) ONS76 cells were transfected with ON-TARGET plus siRNA pools directed against p53 or p21, or with a nontargeting pool (siCon) as a control. 24 h after transfection, cells were divided equally and either left untreated (day 0) or were irradiated with 16 Gy γIR. At days 3 and 5 after irradiation, the cells were harvested and analyzed by western blotting (**C**) and flow cytometrical determination of Zombie-violet-(**D**) and GLF16-positive cells (**E**). For (**D, E**), the mean % of positive cells ± S.D. of three independent experiments is shown. Individual data points are displayed in the bar charts by small filled circles. **p < 0.01, ***p < 0.001. For (**A–C**), the western blots shown are representative of three to four independent experiments. Densitometric analyses of these blots can be found in Supplementary Fig. 2 (**A–F**).

As a next step we analyzed the γIR-mediated responses at the molecular level. Western blotting demonstrated that protein expression of p53 and its target gene p21 was increased in ONS76 cells in a dose-dependent manner (Fig. 3A, Supplementary Fig. 2A, C). Furthermore, Bcl-2 expression was decreased, providing further evidence for a functional p53-activation [35]. In contrast, the already high basal expression of mutated p53 in UW228-3 decreased when the cells were exposed to higher γIR doses (Fig. 3B, Supplementary Fig. 2B). Additionally, low p21-expression was detected (Fig. 3B, Supplementary Fig. 2D), especially when compared to the p21 amounts found in ONS76 at day 5 following exposure to 9 Gy γIR (Fig. 3B, left lane as reference).

We employed siRNAs directed against p53 or p21 to evaluate their functional role in γIR-induced senescence of ONS76 cells. The successful p53 knock-down also resulted in a strongly-decreased γIR-mediated induction of its target gene, p21 (Fig. 3C, Supplementary Fig. 2E, F). Similarly, p21 siRNA transfection reduced p21 protein amounts as expected. However, p53 expression was increased under these conditions. It was already described by us [36] and others [37, 38] that the absence of p21 leads to elevated p53 stabilization. The achieved p53 or p21 knockdown rates were sufficient to result in substantially more cell death (Fig. 3D) and less senescence induction (Fig. 3E) after 16 Gy γIR.

Taken together, these experiments demonstrate that radiotherapy induces cellular senescence in a p53/p21-dependent manner in p53-wild-type ONS76 cells, whereas apoptosis is the primary response of p53-mutated UW228-3 cells.

### c-Myc-overexpression represses γIR-induced senescence and increases cell death

The proto-oncogene c-Myc is a crucial negative prognosis factor in medulloblastoma [39]. In addition, it inhibits p53 signaling pathways targeting p21 in particular [40–42]. Given that the cell lines used in this study express low levels of c-Myc, we employed previously developed ONS76 and UW228-3 cells that constitutively overexpress (OE) either c-Myc or the fluorochrome mCherry as a control [43] to analyze the impact of c-Myc on γIR-mediated cell fates.

**Fig. 4:**
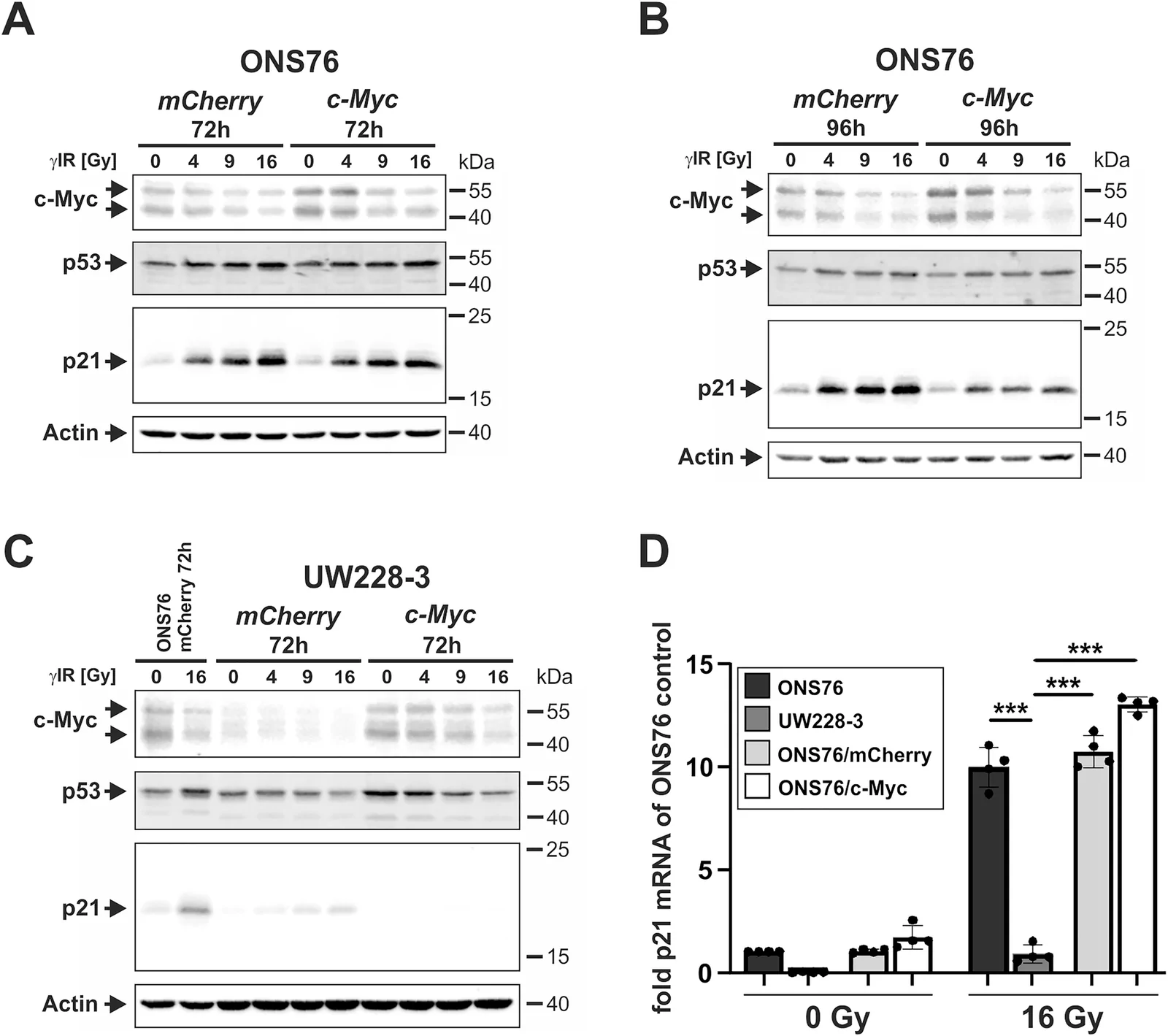
Overexpression of c-Myc results in decreased p21 protein levels after exposure to γIR. ONS76 (**A, B**) and UW228-3 (**C**) cells stably expressing mCherry or c-Myc were exposed to the indicated doses γIR and harvested at day 3 (**A, C**) or 4 (**B**) after irradiation. Protein extracts were analyzed by western blotting for the expression of c-Myc, p53, and p21 as well as actin as a housekeeper control. Please note that the ONS76 samples at day 3 after no treatment or 16 Gy γIR were included on the UW228-3 blot (**C**) for a better comparison of protein expression levels between these two cell lines. This demonstrates the much lower p21 expression in irradiated p53-mutated UW228 cells when compared to irradiated p53 wild-type ONS76 cells. Blots shown are representative for three independent experiments. Densitometric analyses of these blots can be found in Supplementary Figs. 3 and 4. (**D**) qPCR analysis of the relative p21 mRNA expression in parental ONS76 and UW228-3 cells as well as ONS76 cells stably expressing mCherry or c-Myc. All cell lines were left untreated or analyzed at day 5 after exposition to 16 Gy γIR. Expression values were normalized to the untreated ONS76 control cells. Shown is the mean expression ± S.D. of four independent experiments. Individual data points are displayed in the bar charts by small filled circles. ***p < 0.001.

In the ONS76 cell lines, p53 expression was stabilized at similar levels on day 3 and day 4 after γIR regardless of c-Myc-overexpression and the used dose (Fig. 4A, B). However, the γIR-mediated induction of p21 was reduced at day 4 (albeit not at day 3) in the ONS76/c-Myc-OE cells in every single experiment, although because of the observed variances in p21-induction, it was not mathematically significant (Fig. 4B, Supplementary Fig. 3). Similarly, c-Myc-overexpression diminished p21 protein levels in p53-mutated UW228-3 after γIR, even though they exhibited a much lower overall expression of p21 compared to their ONS76 counterparts (Fig. 4C; Supplementary Fig. 3C). This suggests that this p21-reduction is independent of p53 functionality, despite the fact that UW228-3/c-Myc-OE cells expressed higher levels of mutated p53 compared to their mCherry counterparts.

In concordance with the p21 protein data, p21 mRNA levels were strongly upregulated in a p53-dependent manner in the parental ONS76 after irradiation, but not in UW228-3 cells (Fig. 4D). Although c-Myc is a prominent transcriptional regulator of p21 transcription, its mRNA levels were not altered in irradiated c-Myc-overexpressing ONS76 when compared to the ONS76/mCherry control cells (Fig. 4D). This indicates that c-Myc does not affect DNA damage-mediated p21 mRNA transcription but instead modulates either p21 protein production or stability. Taken together, overexpression of c-Myc leads to a decrease in p21 protein but not mRNA levels, irrespective of whether p53 is functional.

**Fig. 5:**
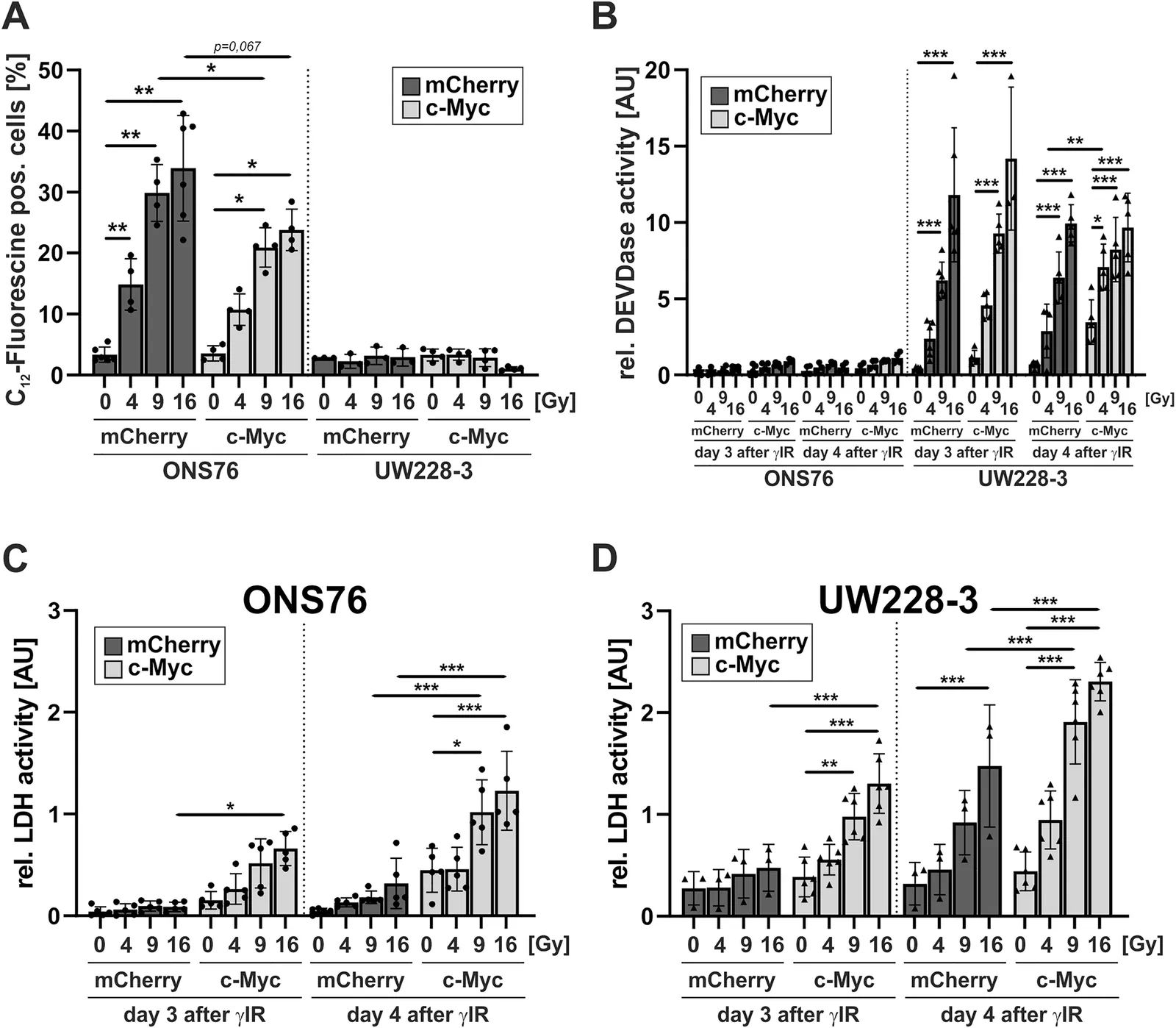
Overexpression of c-Myc results in a p53-independent increase of cell death after γIR. ONS76 (**A–C**) and UW228-3 (**A, B, D**) cells stably expressing mCherry or c-Myc were exposed to different doses γIR and analyzed at day 5 (**A**) or days 3 and 4 (**B–D**) after irradiation. (**A**) Flow cytometric determination of cells positive for senescence-associated beta galactosidase activity. Shown are the mean % of positive cells ± S.D. of three to six independent experiments. (**B**) DEVDase-like caspase activity measured fluorometrically by cleavage of the DEVD-AMC substrate in protein extracts of cells treated with the indicated doses of γIR for the indicated times. (**C, D**) Measured LDH supernatant activity demonstrating cell death induction of cells treated similar as in **B**. For **B–D**, shown are the mean activities/percentages ± S.D. of three to six independent experiments. Individual data points are displayed in the bar charts by small filled circles. *p < 0.05, **p < 0.01, ***p < 0.001.

**Fig. 6:**
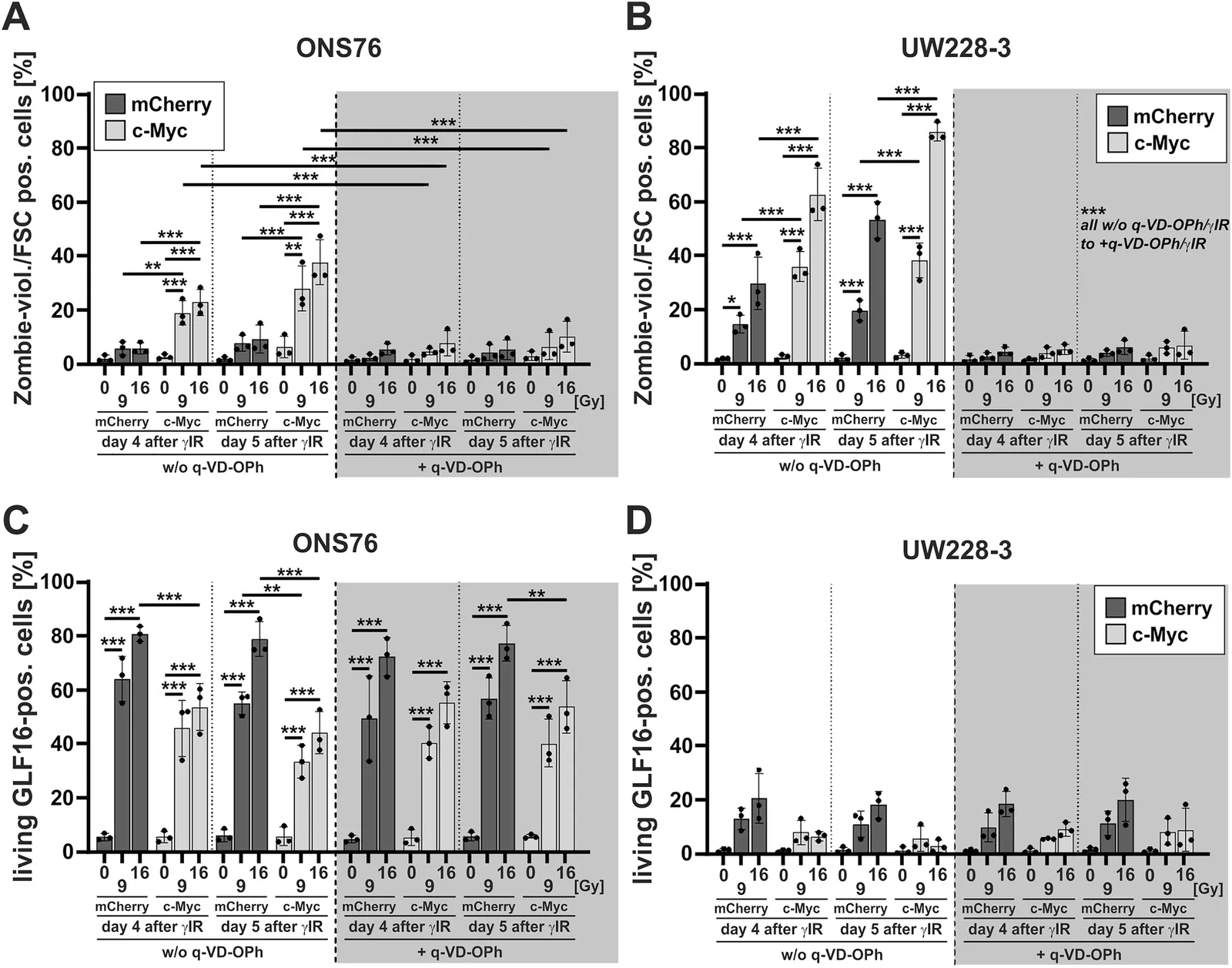
Overexpression of c-Myc results in a decrease of senescence induction and an increase of q-VD-OPh-inhibitable cell death after γIR. ONS76 (**A, C**) and UW228-3 (**B, D**) cells stably expressing mCherry or c-Myc were exposed to different doses γIR in the absence (w/o q-VD-OPh) or the presence ( + 10 µM q-VD-OPh, grey background) of a caspase inhibitor and analyzed at days 4 and 5 after irradiation. Cells positive for the amine-reactive dye Zombie-violet (**A, B**) and the lipofuscin-binder GLF16 (**C, D**) were determined simultaneously by flow cytometry. Shown are the mean % of positive cells ± S.D. of three independent experiments. Individual data points are displayed in the bar charts by small filled circles. *p < 0.05, **p < 0.01, ***p < 0.001.

In concordance with the p21 reduction, visual inspection revealed that a lower number of γIR-exposed ONS76/c-Myc-OE cells exhibited senescent morphologies which in most cases were also less developed (Supplementary Fig. 1B). In addition, more detached and most probably dying cells were visible. Contrary to the ONS76/mCherry samples, many cells displayed a flawed nuclear morphology in form of micro and/or multi-nuclei, indicating that mitotic catastrophe was induced after γIR (Supplementary Fig. 1B, 20× magnification). Flow cytometrical quantification of SA-βG- or GLF16-positive cells demonstrated reduced senescence when c-Myc is overexpressed in γIR-exposed ONS76 cells (Figs. 5A and 6C, D). In UW228-3, whose parental cells already did not undergo senescence after γIR, c-Myc-OE had no additional impact. However, there were significantly more Zombie-violet-labeled cells and more LDH-activities detectable in the supernatant of both c-Myc-overexpressing ONS76 (Figs. 5C and 6A) as well as UW228-3 cells (Figs. 5D and 6B) after γIR when compared to their mCherry expressing counterparts. Furthermore, c-Myc-OE did not result in any changes of γIR-induced caspase activation in both the negative ONS76 nor the positive UW228-3 cells (Fig. 5B), but, in contrast, c-Myc-OE-dependent cell death could be completely inhibited in both lines by using the caspase inhibitor q-VD-OPh (Fig. 6A, B). On the other hand, senescence induction as indicated by a positive GLF16-staining was not affected by caspase-inhibition (Fig. 6C, D). Thereby, independently of the p53 status, c-Myc-OE results in more γIR-mediated cell death, although the exact type is unclear. It is not associated with an increase in caspase activity, but can be abolished by caspase-inhibition. Please note that although it was already shown that c-Myc-expressing cells secrete LDH in a manner not connected to cell death [43], the amount of basal LDH-activity detected in control cells is lower than after γIR treatment. In addition, the LDH-results were confirmed by quantifying cell death using the Zombie-violet marker.

In summary, in our cellular system c-Myc-overexpression resulted in increased irradiation-mediated, q-VD-OPh-inhibitable cell death. In irradiated p53 wild-type cells that usually undergo senescence, c-Myc-OE partially induces cell death instead.

### Therapy-induced senescent ONS76 cells are susceptible toward senolytic treatments

The fact that wild-type p53-expressing ONS76 undergo senescence after γIR exposure allows novel therapeutic approaches. Small molecule inhibitors specifically targeting senescent cells, so-called senolytics, represent exciting novel compounds that could improve anti-cancer therapy by drastically reducing the occurrence of side effects and tumor relapse [23]. We therefore tested if γIR-induced senescent ONS76 cells are susceptible toward such a treatment. Irradiated UW228-3 cells have not been included in these experiments, because they do not become senescent and undergo apoptotic cell death beforehand.

**Fig. 7:**
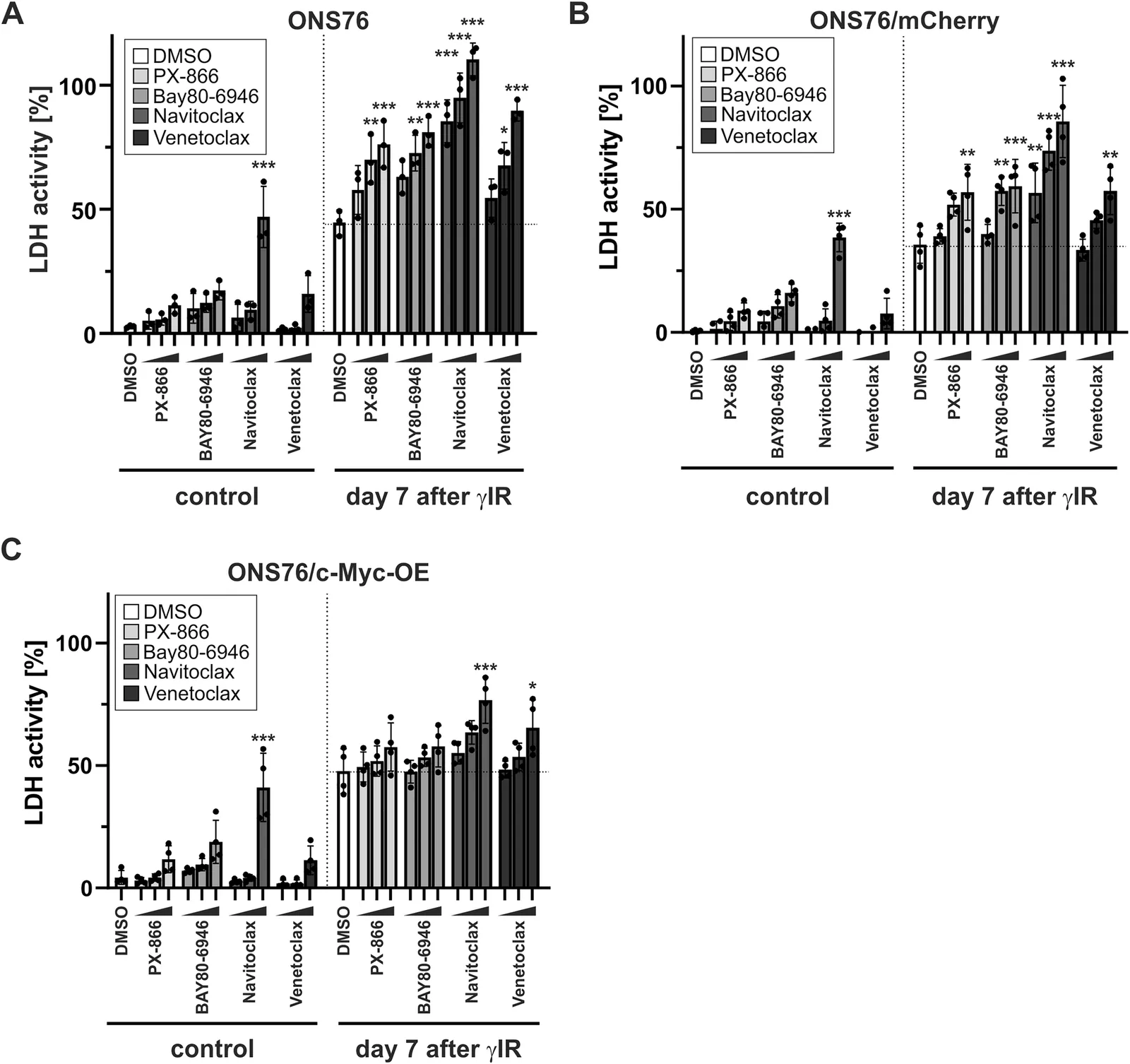
Senolytic substances efficiently eliminate irradiated senescent ONS76 cells, whereas irradiated ONS76/c-Myc cells are more resistant toward such a treatment. Parental ONS76 (**A**) as well as cells stably expressing mCherry (**B**) or c-Myc (**C**) were exposed to 20 Gy γIR to induce senescence. Please note that such a high dose was used to ensure the generation of a full senescent population without any proliferating cells. At day 7 after γIR the cells were treated with varying concentrations of the senolytic substances PX-866, Bay80-6946, Navitoclax, and Venetoclax (all at 3/10/30 µM). 24 h later supernatants were collected and analyzed for LDH activity indicating cell death. Samples treated with Triton-X 100 as a 100% positive control were used to calculate % cell death whereas DMSO-treated cells were used as a solvent control. Shown are the mean % activities ± S.D. of three to four independent experiments. Individual data points are displayed in the bar charts by small filled circles. *p < 0.05, **p < 0.01, ***p < 0.001.

For this analysis we used an experimental setup employed recently by us [44] in which we exposed “senescent” (day 7 after γIR) and “proliferating” (seeded one day before treatment) cells to DMSO (solvent control), triton X-100 (positive 100% control) and senolytic substances such as the Bcl-2 inhibitors Navitoclax and Venetoclax [45] as well as the Pan-PI3K-inhibitors PX-866 and Bay80-6946 (Copanlisib) [44, 46] in varying concentrations for 24 h. Subsequently, LDH activity was measured in the cell culture supernatant to quantify cell death. All four senolytic substances induce death of senescent cells in a dose-dependent manner (Fig. 7A). However, Navitoclax also eliminates proliferating ONS76 cells at the highest used concentration (30 µM), and therefore only is specific for senescent cells at lower doses (Fig. 7A). This in concordance with the big obstacle of utilizing Navitoclax in vivo, because also healthy non-senescent cells are targeted at higher concentrations, resulting in intolerable toxicities [47]. However, our data demonstrate that senolytics can potentially be used to eliminate radiotherapy-induced senescent medulloblastoma cells.

The proliferating and senescent mCherry-expressing ONS76 cells react toward all tested senolytic substances in a similar manner as their parental cells. All compounds specifically eliminate senescent cells (Fig. 7B), although Navitoclax also targets proliferating cells at the highest used concentration. In contrast, the ONS76/c-Myc-OE cell line, in which irradiation results in a reduced number of senescent cells (Figs. 5 and 6), is much more resistant toward a treatment with senolytics (Fig. 7C). Only Navitoclax, and only at the highest used concentration that also eliminates proliferating cells, has a significant albeit still marginal impact on senescent c-Myc-OE cells when compared to wild-type or mCherry-OE ONS76 cells.

In summary, ONS76 medulloblastoma cells with wild-type p53 and low c-Myc expression undergo therapy-induced senescence after being exposed to γIR. These cells can be efficiently eliminated by the use of senolytic compounds. In contrast, irradiation of cells harboring a p53 mutation (UW228-3) or overexpressing c-Myc (ONS76/c-Myc-OE) results in no or reduced senescent cell numbers.

## Discussion

To the best of our knowledge, our study is the first describing γIR-mediated therapy-induced senescence in medulloblastoma cell lines. Expression of wild-type p53, as displayed by ONS76 cells, is crucial for this form of cellular senescence. Exposure of these cells to γIR results in a typical senescent morphology (Fig. 1; Supplementary Fig. 1) [33], p53 stabilization, increased p21 expression (Fig. 3; Supplementary Fig. 2), elevated lysosomal senescence-associated beta-galactosidase activity (Fig. 2A), and lipofuscin accumulation (Fig. 2E). All these features are observed in the absence of cell death (Fig. 2) and require sufficient levels of p53 and p21 (Fig. 3), clearly demonstrating the senescent status of irradiated ONS76 cells. UW228-3 cells expressing mutated p53, however, do not induce p21 in substantial amounts, which would be essential for DNA damage-mediated senescence [48–51], and are eliminated by γIR-mediated apoptosis.

The occurrence of other forms of senescence in medulloblastoma cells has been successfully identified in several reports. For example, valproic acid [52] or inhibition of citron kinase [53] are powerful inducers of cellular senescence. As another subtype, oncogene-induced senescence represents an important tumor suppressor mechanism which is driven by the overexpression/activation of oncogenes during tumorigenesis [27, 39, 54]. Evasion of this process by either ARF/p16- or p53/p21-signaling pathway inactivation has been shown to be very important for medulloblastoma progression [28]. This demonstrates that cellular senescence represents a default program for medulloblastoma progenitor cells that could also be activated by other triggers, such as DNA damage during therapy, if key molecular players such as p53 remain intact (i.e., if ARF/p16 is mutated during tumorigenesis).

Interestingly, in contrast to other cancer entities, medulloblastoma do not harbor many p53 mutations [55]. On the other hand, p53 mutation is known to be the most important prognostic risk factor for SHH-activated medulloblastoma. Hence, the SHH-activated subtype is divided into two subgroups differentiated only by their p53 wild-type/mutational status, and both require different treatment approaches [2]. Based on their 5-year-overall survival the p53-mutated SHH-activated subgroup is classified as high-risk disease (41%), whereas the prognosis in p53 wild-type tumors is much better (81%, intermediate risk) [56–58]. One important difference between these two subgroups, especially in the context of radiotherapy, is the induction of DNA damage-mediated cellular senescence (p53 wild-type) vs cell death/apoptosis (p53-mut). It is important to note that this distinction is not detected in the usually performed cytotoxicity assays that are based on a proliferation readout. Our findings to use radiotherapy in concert with senolytic compounds would therefore primarily be feasible in the intermediate risk group with superior outcome. However, employing a senolytic approach could further improve survival rates and potentially decrease side effects such as impaired neurocognition due to lower required radiation doses and the elimination of senescent cells.

c-Myc-overexpression resulted in a reduced formation of the senescent phenotype and abolished the effect of senolytic substances. In primary healthy cells, c-Myc-activation results in oncogene-mediated senescence [39, 40]. However, the herein used cell lines have evaded this cell fate by mutation of the ARF signaling pathway (ONS76) or p53 (UW228-3) during tumorigenesis [28]. c-Myc and the p21 represent two antagonists, battling each other at multiple fronts. For example, c-Myc diminishes p21 expression [40–42], mostly on the transcriptional level in a p53-independent manner, and p21 decreases c-Myc transcriptional activity [49, 59]. Because p21 is essential for the induction of DNA damage-induced senescence [48], this highlights a delicate balance between these two proteins that determines whether senescence is induced. Thus, it is not very surprising that c-Myc-overexpression negatively impacted both p21 protein expression (but not its mRNA) and γIR-mediated senescence in our hands (Figs. 4–6). Consequently, irradiated c-Myc-overexpressing ONS76 cells were more resistant toward senolytic treatment (Fig. 7). With JQ1, a novel approach is undertaken to target c-Myc-activation in medulloblastoma by inhibiting the bromodomain protein BRD4 [60]. In chondrosarcoma cells, JQ1 induced premature senescence and apoptosis, demonstrating that c-Myc inhibition freed and activated the p53/p21 signaling axis [61, 62]. It would be very interesting to test if JQ1 impacts wild-type p53/c-Myc-high medulloblastoma cells in a similar manner and increases the amount of γIR-induced senescence, in turn rendering them susceptible toward senolytic treatment.

The elimination of senescent cells using senolytics represents a promising approach for the treatment of age-related pathologies and cancer [23, 63]. Our results (Fig. 7) demonstrate that senescent medulloblastoma cells are killed by senolytics such as the FDA-approved [46] Bay80-6946 [44] or the Bcl-2-inhibitor Navitoclax [45]. Similar to the observed response to whole brain irradiation [64, 65], the standard CSI-therapy of medulloblastoma surely also results in the generation of senescent cells and, although it is associated with severe side effects, is so far irreplaceable for a successful anti-cancer treatment [9, 10]. It is therefore tempting to speculate that CSI-induced senescent cells and their secretion of inflammatory factors in form of the SASP support or even cause negative outcomes [66], and that an additional treatment with senolytics could potentially inhibit these fatal processes. For glioblastoma, it has already been shown that (a) p21-dependent senescence induction benefits tumor growth and (b) the timely elimination of therapy-induced senescent cells using Navitoclax attenuates recurrence [67], demonstrating the high potential of such an approach. As another point in favor for the use of senolytic compounds in cancer treatment, cellular senescence can be triggered at lower Gy-doses than cell death [68]. A successful combination of senolytics with CSI could thereby reduce the required radiation exposure for patients while achieving similar tumor control.

Taken together, although our results were obtained in cell lines, they indicate that SHH-activated medulloblastoma cells with functional wild-type p53 and low c-Myc-expression undergo cellular senescence after γIR exposure. Subsequently, these senescent cells can be efficiently removed by senolytic substances. Translating this senolytic option to the clinic could potentially benefit the oncological outcome and inhibit the occurrence of severe short- and long-term side effects.

## Materials and methods

### Cell lines, treatments, and reagents

The SHH-activated medulloblastoma cell lines ONS76 (p53 wild-type) and UW228-3 (p53-mutated) were a kind gift of P. Landgraf (University of Cologne, Germany). In addition, we used previously generated cells stably transfected with the oncogene MYC or the fluorochrome mCherry as a control [43]. All cell lines were cultivated in Dulbecco’s Modified Eagle’s Medium containing GlutaMAX, 4.5 g/l D-glucose and sodium pyruvate (DMEM, Gibco, Waltham, MA, USA). In addition, the medium was supplemented with 10% heat-inactivated fetal bovine serum (FBS Supreme, PAN-Biotech GmbH, Aldenbach, Germany). Cell lines were authenticated by the Multiplex human cell line Authentication Test (MCA; Multiplexion GmbH, Heidelberg, Germany) and regularly thawed (every 2–6 months) from these controlled batches. Furthermore, cultured cells were routinely tested for mycoplasma contamination using the Venor®GeM detection kit (Minverva Biolabs, Berlin, Germany). Cells were exposed to γIR using the Gulmay RS225 irradiation system (Xstrahl GmbH, Ratingen, Germany) at 15 mA and 150 kV (for doses < 10 Gy) or 175 kV (for doses > 10 Gy). We obtained the PI3K inhibitors PX-866 and Bay80-6946 as well as the fluorogenic caspase-3 substrate DEVD-AMC (N-acetyl-Asp-Glu-Val-Asp-aminomethylcoumarin) from Biomol GmbH (Hamburg, Germany), whereas we got the BH3 mimetics and Bcl-2-family inhibitors Navitoclax and Venetoclax from Selleckchem (Cologne, Germany). From Sigma Aldrich (Merck KGaA, Darmstadt, Germany) we acquired X-gal (5-bromo-4-chloro-3-indolyl-β-D-galactopyranoside), DTT, the protease inhibitors PMSF, aprotinin, leupeptin, pepstatin, and the phosphatase inhibitors sodium ortho-vanadate and sodium pyrophosphate. DMSO was purchased from Applichem GmbH (Darmstadt, Germany) and the senescence-associated beta-galactosidase substrate C_12_-FDG (5-Dodecanoylaminofluorescein di-beta-D-Galactopyranoside) was from MedChemExpress (Sollentuna, Sweden). The pan-caspase-inhibitor q-VD-OPh was from MP Biomedicals (Irvine, CA, USA).

### Antibodies

The mouse monoclonal p53 antibody (clone Ab-6; #OP43; AB_213405) was obtained from Calbiochem (Bad Soden, Germany), whereas we used the rabbit monoclonal p21 (clone 12D1; #2947; AB_823586) and c-Myc (clone D3N8F; #13987; AB_2631168) antibodies from Cell Signaling Technology (Leiden, Netherlands). From Santa Cruz (Heidelberg, Germany) was the mouse monoclonal Bcl-2 antibody (clone C-2; # sc-7382; AB_626736). The mouse monoclonal antibody directed against the loading control protein beta-actin (clone AC74; #A5316; AB_476743) was purchased from Sigma-Aldrich. All infrared fluorescence-labeled secondary antibodies were bought from LI-COR Biosciences (Lincoln, NB, USA).

### Transfection of siRNA SMART pools

ONS76 cells were transfected with ON-TARGET siRNA SMART pools directed against p53, p21, or with a non-targeting control (siCon) in 6 cm-dishes using DharmaFECT 1 according to the manufacturer’s instruction (Dharmacon, Horizon Discovery, Cambridge, UK). 24 h post transfection, the cells were trypsinized and divided equally for later western blot and flow cytometric analyses. After reattachment (4–6 h), the cells were left untreated (day 0) or exposed to 16 Gy γIR. After incubation for 3–5 days, the cells were harvested and subsequently analysed. Western Blotting was used to assess the efficacy of protein knockdown, whereas a simultaneous staining of Zombie-violet and GLF16 followed by flow cytometry was employed to determine the functional impact of the knock-down.

### Colorimetric detection of senescence-associated beta-galactosidase activity in γ-irradiated cells

Cells were exposed to γIR in 6-well plates. At the indicated times, cells were fixed for 5 min at room temperature with 0.2% glutaraldehyde and 2% paraformaldehyde. After two washings with PBS, the fixed cells were incubated at 37 °C overnight for 14 h in a staining buffer [69] containing X-Gal at a suboptimal pH of 6.0, only allowing the detection of the massively increased senescence-associated beta-galactosidase activity. Upon X-Gal cleavage, cells display a strong and clearly visible blue color. The staining was stopped by repeated washes with PBS and storage of the well plates at 4 °C. Pictures were taken with 10× or 20× magnification as indicated using the Zeiss Axio Vert.A1 microscope and the associated AxioVision Software (Carl Zeiss MicroImaging GmbH, Göttingen, Germany).

### Flow cytometric determination of senescence-associated beta-galactosidase activity in γ-irradiated cells

To quantify the amount of cells positive for senescence-associated beta-galactosidase activity, we modified a flow cytometric protocol [70] using the substrate C_12_-FDG. At day 5 after γIR, the cell culture supernatant was transferred from a 12 well plate into a 15 ml tube and centrifuged to pellet the detached dead cells. During this procedure, 500 µl medium supplemented with 100 nM Bafilomycin A1 (BafA1) was given to remaining adherent cells in the well. After the centrifugation, the supernatant in the tubes was aspirated. Subsequently, the pellet of dead cells was resuspended in 500 µl BafA1-medium and transferred back into their corresponding original well for further incubation at 37 °C. This procedure permits the inclusion of dead cells and thus, the determination of the correct %-ratio of living, potentially senescent cells to dead cells. After 1 h, each well was supplemented with 16.5 µl of 2 mM C_12_-FDG, mixed thoroughly and again incubated for another 2 h at 37 °C. Cells were harvested into FACS tubes together with their supernatant by trypsinization and washed two times with PBS. Finally, the samples were resuspended in 200 µl PBS and analyzed using a FACS-Verse flow cytometer (BD Biosciences) and the FACSuite analysis software. Using a histogram, the number of cells positive for C_12_-fluorescine when opposed to negative proliferating cells was determined.

### Simultaneous flow cytometric determination of senescence and cell death using the Zombie-violet and GLF16 dye

To successfully determine the percentages of senescent and dead cells after irradiation, we combined protocols for the staining with the amine-reactive dye Zombie-violet (BioLegend, Amsterdam, Netherlands) with the recently published labeling protocol with the lipofuscin-binder GLF16 (TargetMol, Linz, Austria) [34, 71]. At the indicated times after irradiation, the cells were harvested together with their supernatants at room temperature. After washing with PBS, the cells were labeled with 100 µl Zombie staining buffer for 15 min on ice and in the dark, as described by the manufacture’s instruction (Biolegend). The reaction was stopped by addition of 1 ml PBS supplemented with 4% BSA. After another washing step with pure PBS, the cells were fixed in 200 µl 4% formaldehyde for 15 min at room temperature. After another washing step, the cells were stored in 1 ml 90% methanol at −20 °C until measurement. The methanol was subsequently removed by washing with PBS and the cells stained for lipofuscin accumulation by using the fluorescent Sudan Black B-analogue GLF16 (100 µl of a 10 µg/ml solution in dilution buffer (2.5% DMSO/2.5% Tween-20 in PBS)) for 8 min at room temperature and in the dark. The staining was stopped by the addition of 2 ml dilution buffer and an additional washing step with 1 ml dilution buffer. Subsequently, the cells were resuspended in 150 µl PBS and analyzed using a FACS-Verse flow cytometer (BD Biosciences) and the FACSuite analysis software. Using a V450 (Zombie-violet)/FSC dot plot, living and dead cells were identified (dead cells become fragmentated and Zombie-violet positive). GLF16 positive cells were determined using a histogram and the APC-Cy7 channel. Senescent cells were defined to be “living” (non-Zombie-violet pos./FSC) and GLF16 positive.

### Quantification of cell death by measuring LDH activity in the cell culture supernatant

Cell death is usually associated with a loss of plasma membrane integrity and the release of cytoplasmatic lactate dehydrogenase (LDH) into the cell culture supernatant. This experimental approach offers the big advantage of measuring cell death in senescent cells without the need to handle these delicate enlarged cells that unfortunately easily die upon forced detachment by trypsinization and pipetting. In addition, this assay can be performed side-by-side with other analyses such as Western blotting. We used the cytotoxicity detection kit (LDH) from Roche (Mannheim, Germany) according to the manufacturer’s instructions. In short, at the indicated times after γIR exposure, 100 µl of the cell culture supernatant was transferred to a 96-well plate and stored at 4 °C. When all samples of one experimental setup were collected, 100 µl reaction mixture from the kit was added to each well. Absorbance at 490 nm, resembling LDH activity, was measured for 30 min in 3-min-intervals at room temperature in a Tecan Infinite M200 microplate reader. As a reference for unspecific signals, the absorbance at 690 nm was also determined. Data are either presented as arbitrary units (AU) or as % of total LDH activity (with 100% activity calculated from control wells that were treated and lysed with 1% Triton-X 100 for 30 min).

### Cell extracts and western blotting

Cells were lysed for 30 min at 4 °C in NP40 lysis buffer containing 1% NP-40, 50 mM Tris-HCl at pH 7.4, 150 mM NaCl. The lysis buffer was freshly supplemented with 1 mM DTT, the protease inhibitors leupeptin, pepstatin, aprotinin (all at 10 µg/ml), PMSF as well as the phosphatase inhibitors sodium orthovanadate and sodium pyrophosphate (all at 1 mM). After a 30 min centrifugation to eliminate insoluble cell debris, protein concentration was measured using the Bradford protein assay (BioRad, München, Germany). 30 µg cellular extract per lane was subjected to SDS-PAGE and subsequently transferred onto low-infrared-emitting PVDF membranes (Millipore, Schwalbach, Germany) by western blotting. After blocking the membrane with 4% nonfat dry milk in PBS/T (PBS/0.2% Tween20), the membranes were incubated overnight with the primary antibodies and, after several washing steps with PBS/T, additionally incubated with infrared fluorochrome-labeled species-specific secondary antibodies. Proteins on the membranes were visualized by scanning with the LI-COR Odyssey FC and densitometrically analyzed with the Image Studio V5.2 software.

### Quantification of caspase-3-like DEVDase activities in cellular extracts

Fluorometric determination of caspase activities was performed as described [72]. In short, 50 µg cellular extracts were mixed in a black 96-well plate with a HEPES-based caspase substrate buffer at pH 7.3 that was freshly supplemented with the fluorogenic substrate Ac-DEVD-AMC (50 µM) and 10 mM DTT. Caspase activities resulting in the release of AMC were measured in a Tecan Infinite M200 microplate reader at an emission of 346 nm and an excitation of 442 nm for 3 h at 37 °C in 10-min intervals.

### Isolation of cellular mRNA and quantification by real-time PCR

Cellular mRNA was isolated from harvested cells using the RNeasy Mini Kit from Qiagen (Hilden, Germany) according to the manufacturer’s instructions. Subsequently, 1 µg RNA was transcribed into the cDNA using the High-Capacity cDNA Kit (ThermoFisher Scientific, Darmstadt, Germany). Afterwards, p21 (CDKN1A) mRNA levels in 25 ng cDNA of each sample were assessed with a specific TaqMan probe (ID# Hs99999142_m1) in an ABI 7300 cycler (Applied Biosystems) using actin mRNA (ID# Hs99999903_m1) as an endogenous control. Relative expression values were calculated using the 2^(Δ(ΔCt))^ method and normalized to untreated ONS76 cells.

### Statistical analyses

After checking the obtained data for normal distribution, a nonparametric *t*-test or a one-way analysis of variance (ANOVA) followed by a post hoc Tukey’s multiple comparisons test (for three or more groups) was performed using GraphPad Prism 8 if applicable. *p < 0.05, **p < 0.01, ***p < 0.001. Every analyzed experiment included negative and positive controls.

## Supplementary information

Supplemental Figures including legends

Supplemental Raw WB Data

## Data Availability

The data generated in this study are available upon request from the corresponding author.
