# Supplementary material for: p53 wild-type/low c-Myc-expressing ONS76 pediatric medulloblastoma cells become senescent after gamma-irradiation and can be eliminated by senolytic substances
Source: Cell Death Discov. 2026 Oct 8;12:410. doi: 10.1038/s41420-026-03404-4 (PMC13649648; doi:10.1038/s41420-026-03404-4)
Supplement: Supplementary file 1 [file 41420_2026_3404_MOESM1_ESM.pdf]

Suppl. Figure 1

Sonntag et al. 2026

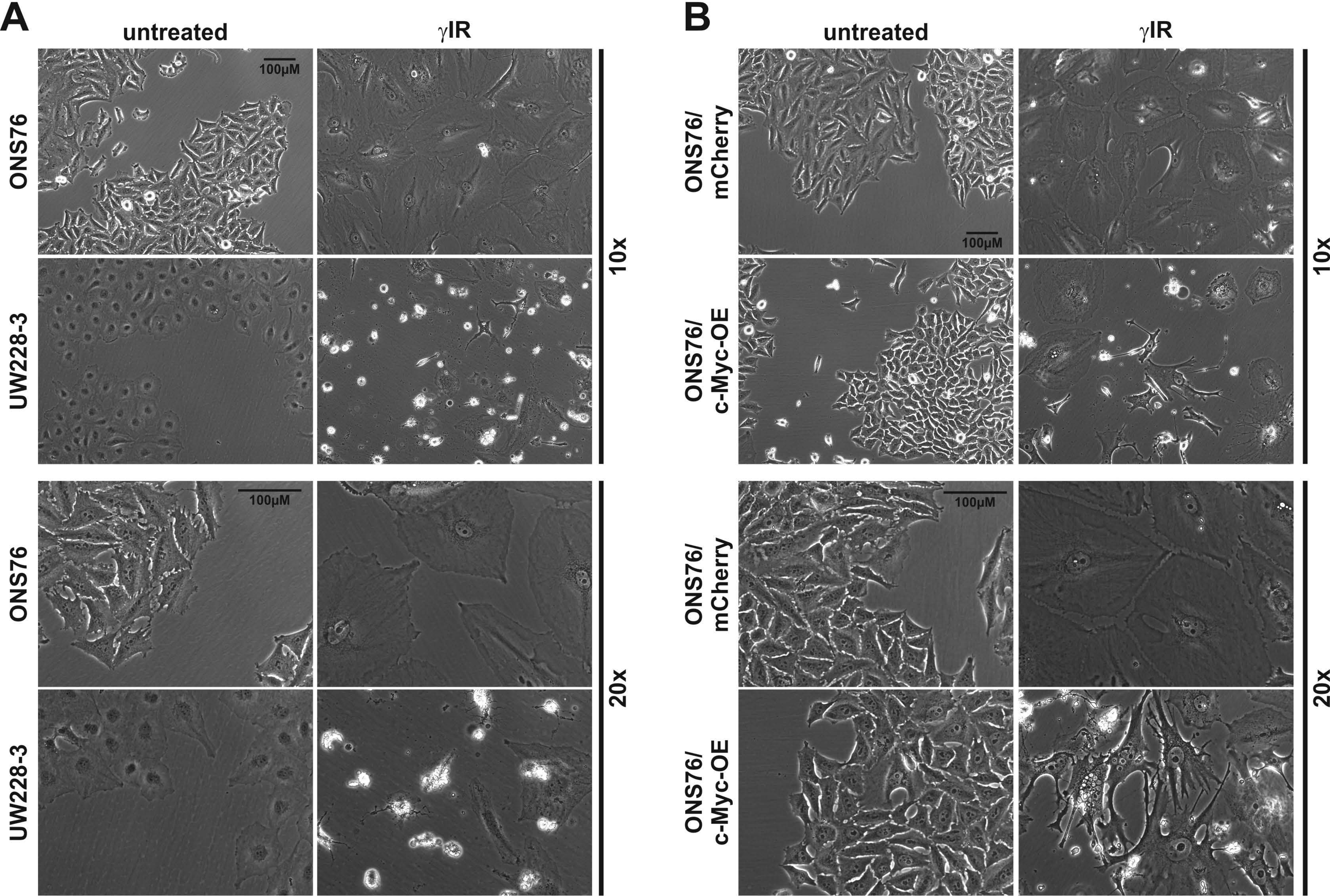

Supplementary Figure 2

Sonntag et al. 2026

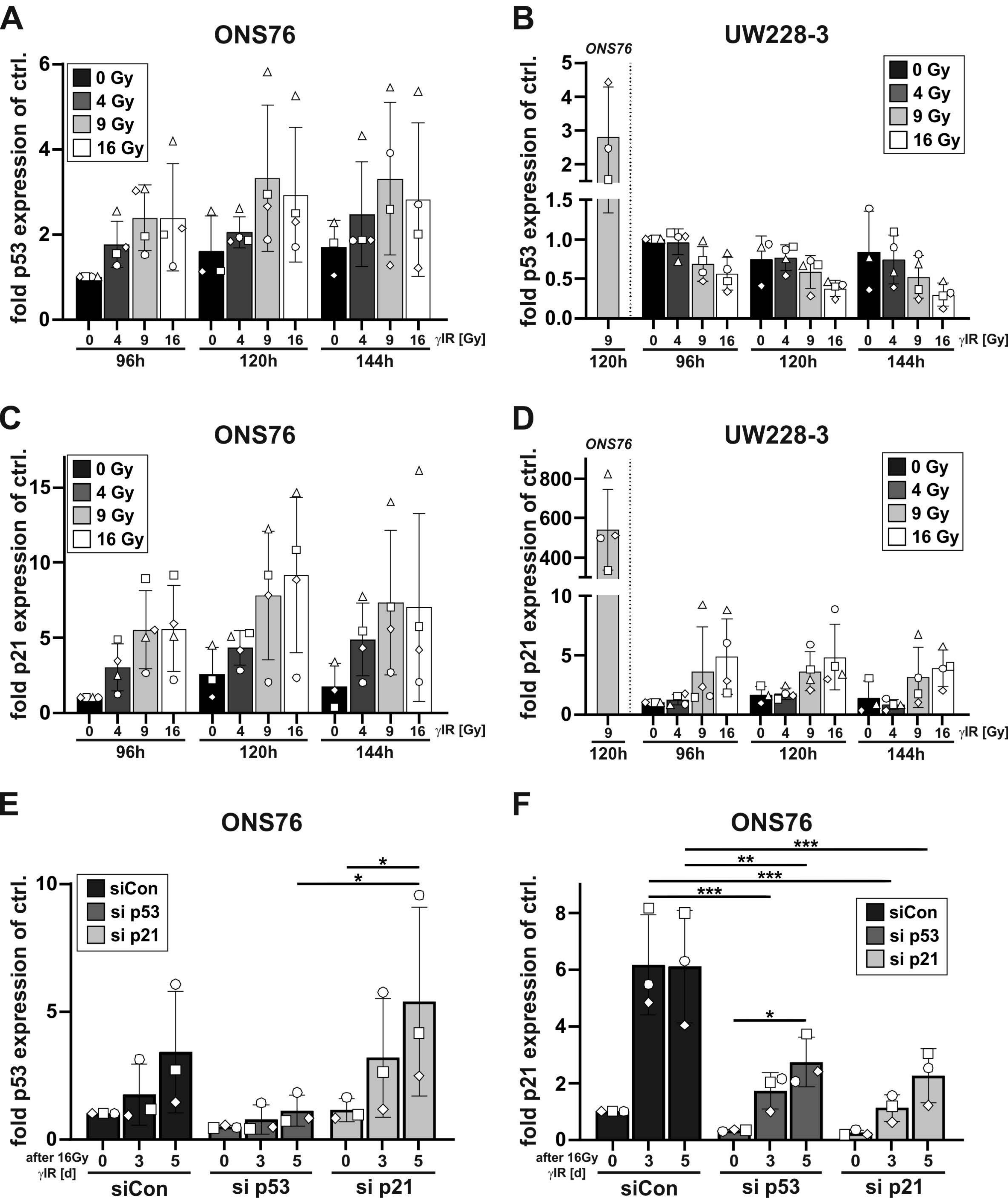

Supplementary Figure 3

Sonntag et al. 2026

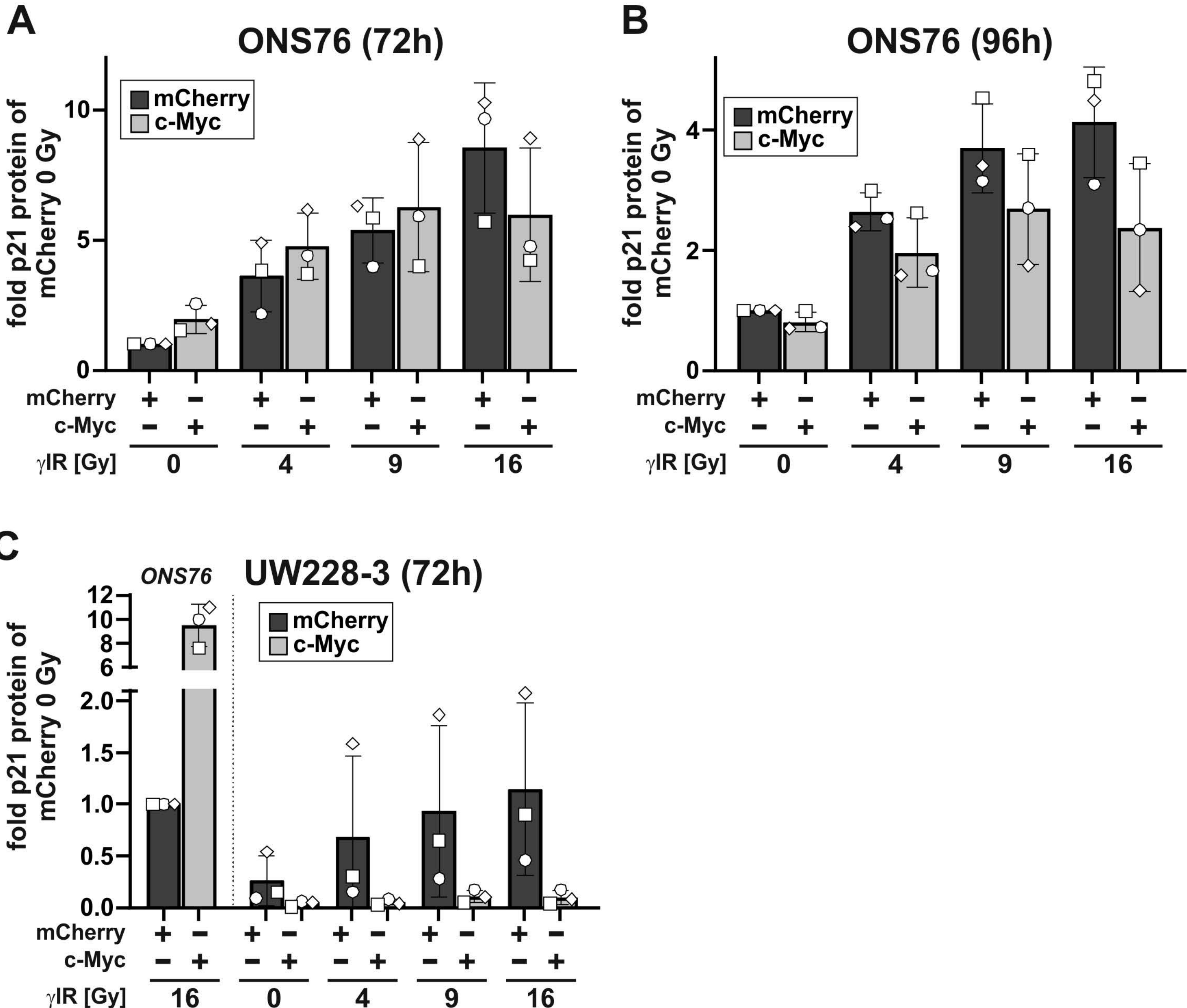

Supplementary Figure 4

Sonntag et al. 2026

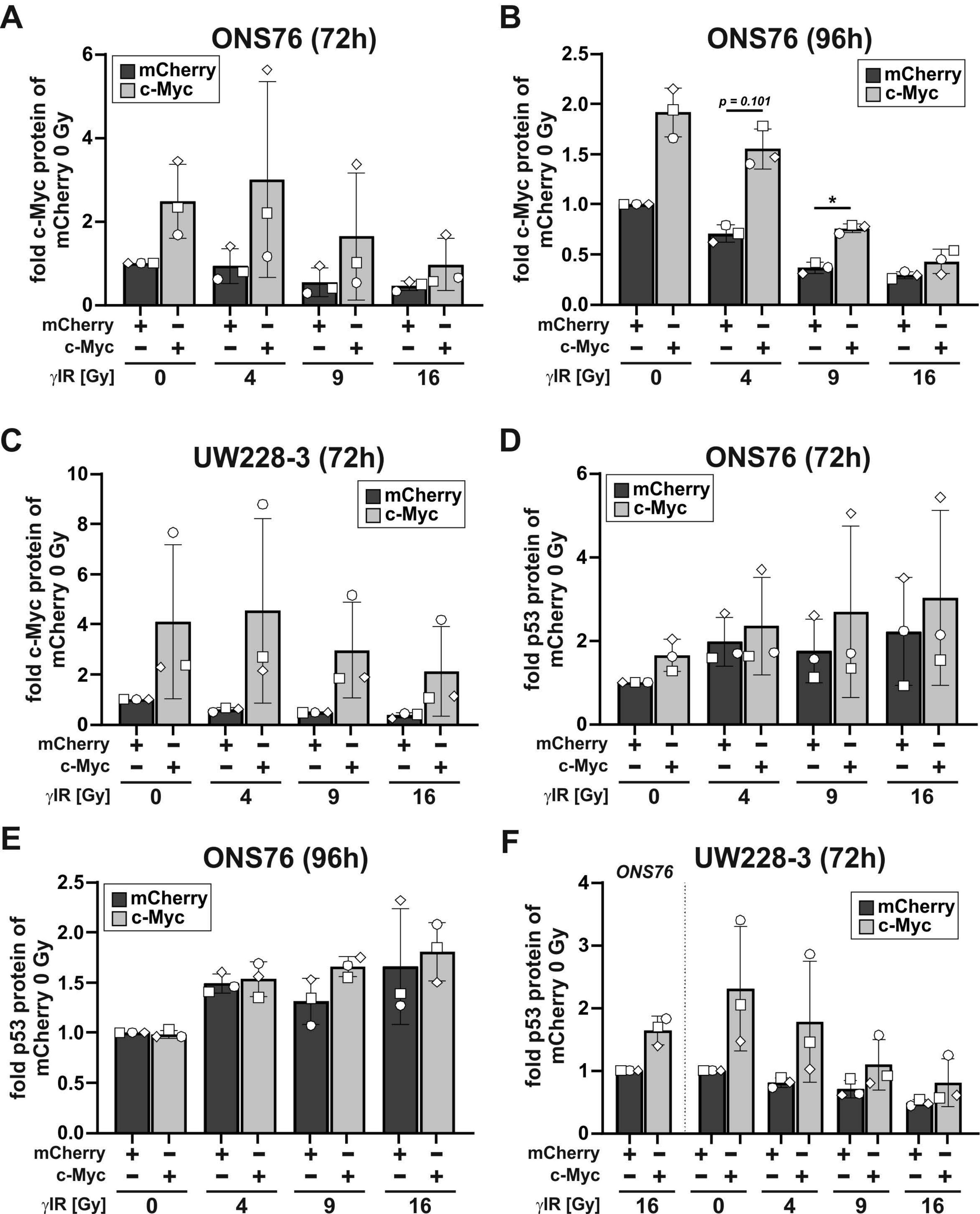

***Supplemental Figure 1.* In contrast to their parental ONS76 and their mCherry expressing counterparts, both c-Myc-overexpressing ONS76 and p53-mutated UW228-3 cells do not display a senescent cellular and nuclear morphology after irradiation.** ONS76 and UW228-3 (**A**) as well as mCherry or c-Myc-overexpressing ONS76 cells (**B**) were exposed to 16Gy γIR or left untreated and analyzed microscopically at day 5 after irradiation. Phase contrast (Ph1) pictures were taken at 10x (upper panels) and 20x magnification (lower panels). Shown are representative pictures from at least three independent experiments. The included size bar depicts 100 µm. Irradiated parental ONS76 as well as mCherry-expressing cells display senescent morphological alterations associated with senescence, such as a flattened cellular appearance and enlarged but still intact nuclei. In contrast, a substantial portion of irradiated UW228 and the c-Myc-expressing ONS76 cells are detached and fragmentated, indicating cell death. In addition, only a few adherent cells are visibly enlarged and several (c-Myc-OE ONS76) to nearly all (UW228-3) cells show aberrant nuclear alterations, e.g., multi- and/or micro-nuclei. Please note that in contrast to Figure 1, the shown pictures were taken under cell culture conditions and not from fixed cells. Thereby, dying/dead cells are still visible in the supernatant.

***Supplemental Figure 2.* γIR-stabilized p53 and γIR-upregulated p21 can be successfully knocked down using specific ON-Target plus siRNA smart pools.** Densitometric analyses of the western blots shown in figure 3. Each panel (**A-F**) depicts the combined data from all experiments that were represented in the corresponding panel in figure 3. The signal of the p53 or p21 antibody was normalized to the signal of the corresponding actin housekeeping protein. Shown is the mean of three to four independent experiments +/- S.D. of the calculated fold expression of p53 or p21 compared to the ONS76 (**A, C, E-F**) or UW228-3 (**B, D**) control sample. Different shapes (empty square, circle, diamond, triangle) display the obtained values from every single experiment. * $p < 0.05$, ** $p < 0.01$, *** $p < 0.001$. Please note that small

fluctuations of low p53 or p21 basal expression in the control sample of one certain experiment can result in bigger variances of calculated fold p53- or p21-expression of irradiated samples, as can be observed by comparing the single experiment data. Because of the high variance, mathematical significant differences were not always achieved, although similar tendencies were observable, e.g., p53 and p21 expression is increased in a dose-dependent manner in ONS76 cells in every single experiment (**A, C**). Likewise, p53 and p21 protein expression is successfully knocked down in every single experiment (**E, F**).

Supplemental Figure 3. **Overexpression of c-Myc results in a tendency of decreased p21 protein levels after exposure to γIR.** Densitometric analyses of the p21 protein western blots shown in figure 4. Each panel (**A-C**) depicts the combined data from all experiments that were represented in the corresponding panel in figure 4. The signal of the p21 antibody was normalized to the signal of the corresponding actin housekeeping protein. Shown is the mean of three independent experiments +/- S.D. of the calculated fold expression of p21 compared to the ONS76 (**A, B**) or UW228-3 mCherry (**C**) control sample, respectively. Different shapes (square, circle, diamond) display the obtained values from every single experiment. Please note that small fluctuations of low p21 basal expression in the control sample of one experiment can result in bigger variances of calculated fold p21 expression of irradiated samples, as can be observed by comparing the single experiment data. Because of the high variance, no mathematical significant differences between mCherry and c-Myc-OE cells could be detected, although similar tendencies were observed, e.g., ONS76/c-Myc-OE cells displayed lower p21 protein expression after 96 hours in every single experiment.

Supplemental Figure 4. **Densitometric analyses of c-Myc and p53 protein expression in stably mCherry or c-Myc-transfected ONS76 or UW228-3 cells after γIR.** Densitometric analyses of c-Myc (**A-C**) and p53 (**D-F**) protein western blots shown in figure 4. Each panel (**A-F**) depicts the combined data from all experiments that were represented in the

corresponding panels in figure 4. The signal of the c-Myc or p53 antibody was normalized to the signal of the corresponding actin housekeeping protein. Shown is the mean of three independent experiments +/- S.D. of the calculated fold expression of c-Myc compared to the ONS76 (**A, B, D, E**) or UW228-3 mCherry (**C, F**) control sample. Different shapes (square, circle, diamond) display the obtained values from every single experiment. * $p < 0.05$. Although c-Myc-OE is only mathematically significant under one condition because of the inherent variance, the single experiment data demonstrate that c-Myc was overexpressed was in every single performed experiment.
