# Supplementary material for: p53 wild-type/low c-Myc-expressing ONS76 pediatric medulloblastoma cells become senescent after gamma-irradiation and can be eliminated by senolytic substances
Source: Cell Death Discov. 2026 Oct 8;12:410. doi: 10.1038/s41420-026-03404-4 (PMC13649648; doi:10.1038/s41420-026-03404-4)
Supplement: Supplementary file 2 [file 41420_2026_3404_MOESM2_ESM.pdf]

Supplemental Raw Data
Western Blot

Sonntag et al. 2026

Figure 3A

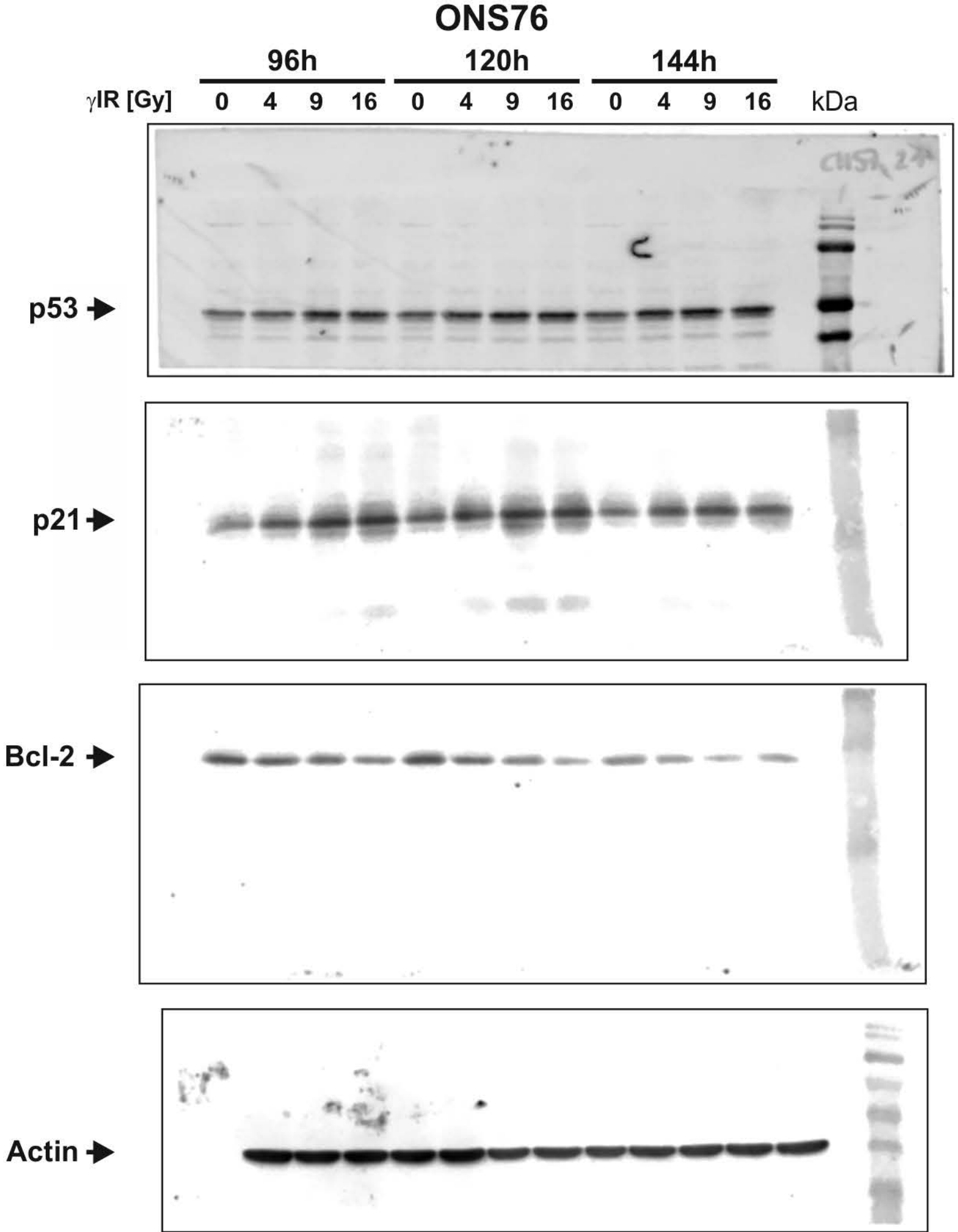

Figure 3B

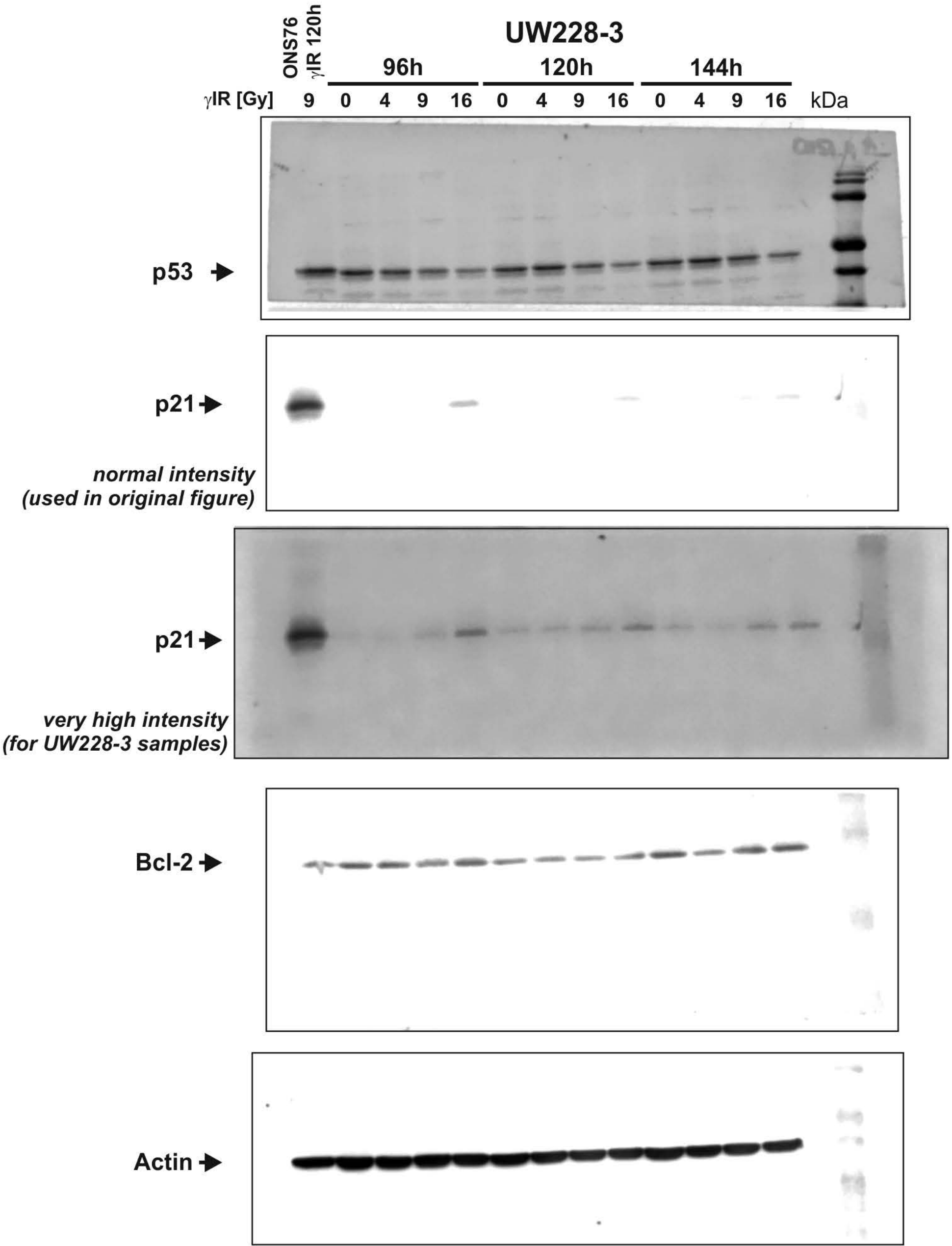
UW228-3
ONS76
γIR 120h
96h
120h
144h
γIR [Gy]
9
0
4
9
16
0
4
9
16
0
4
9
16
kDa
p53
p21
normal intensity
(used in original figure)
p21
very high intensity
(for UW228-3 samples)
Bcl-2
Actin

Figure 3C

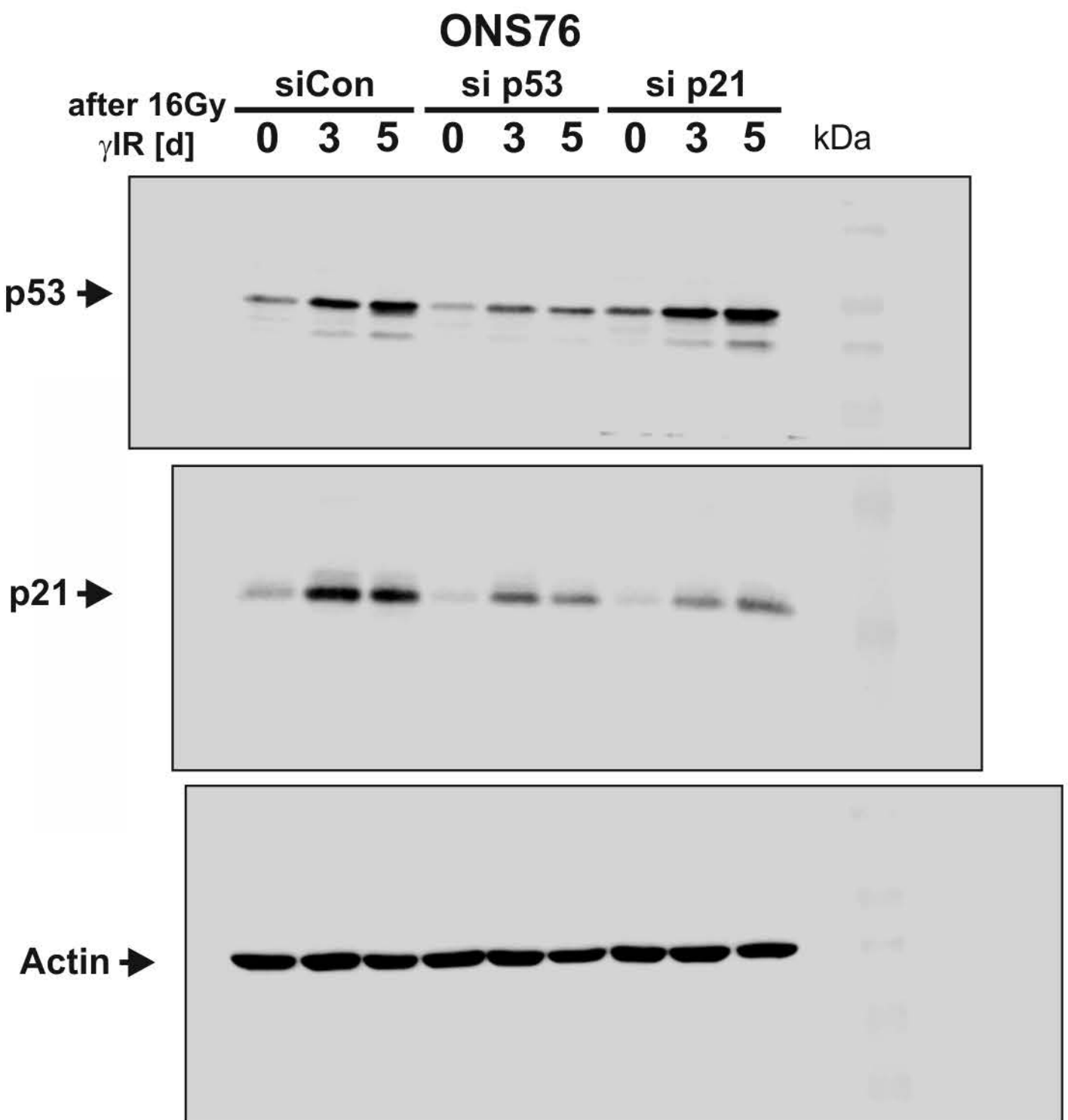
ONS76
after 16Gy γIR [d]
siCon
si p53
si p21
0 3 5 0 3 5 0 3 5
kDa
p53
p21
Actin

Figure 4A

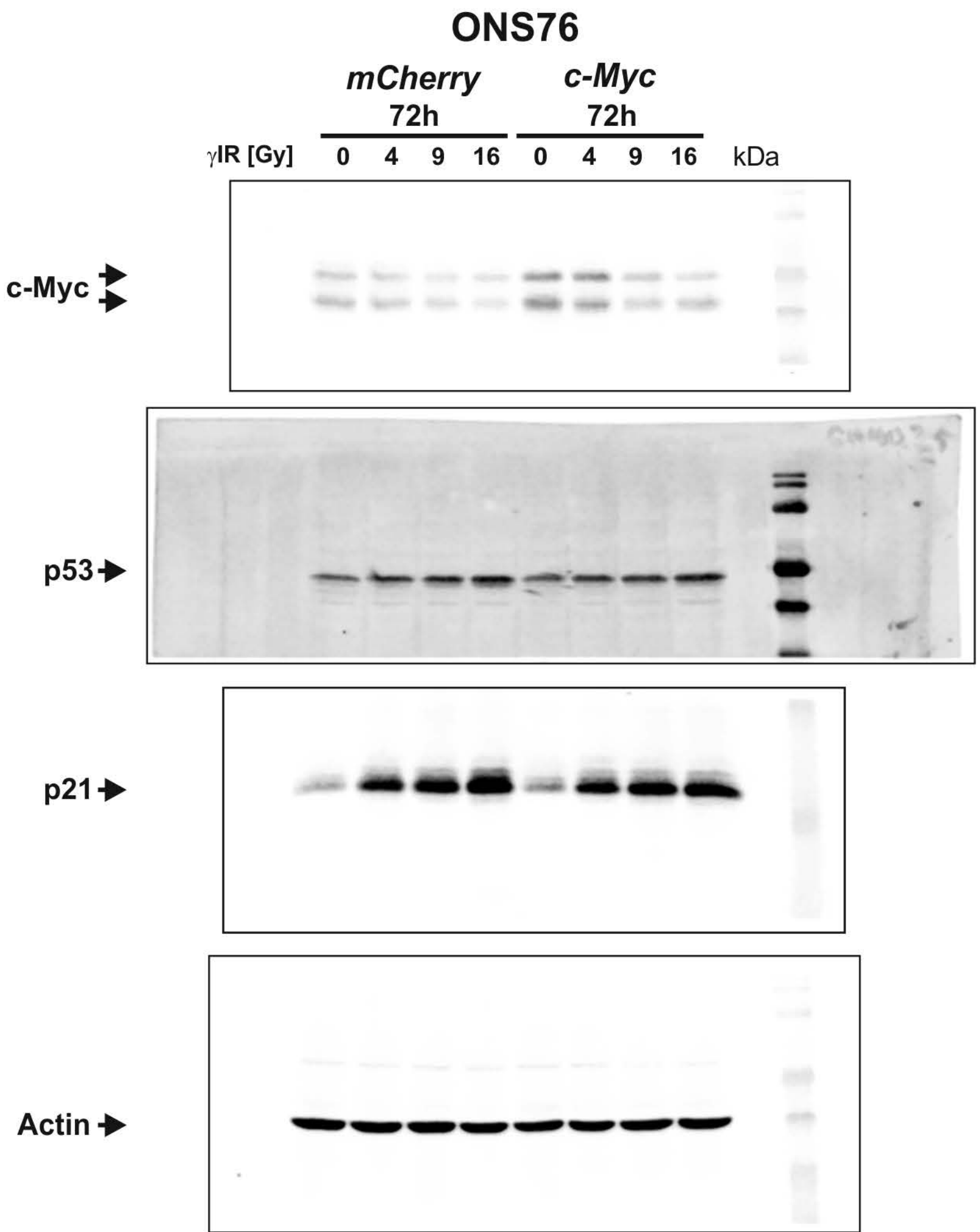
ONS76
mCherry
72h
c-Myc
72h
γIR [Gy]
0
4
9
16
0
4
9
16
kDa
c-Myc
p53
p21
Actin

Figure 4B

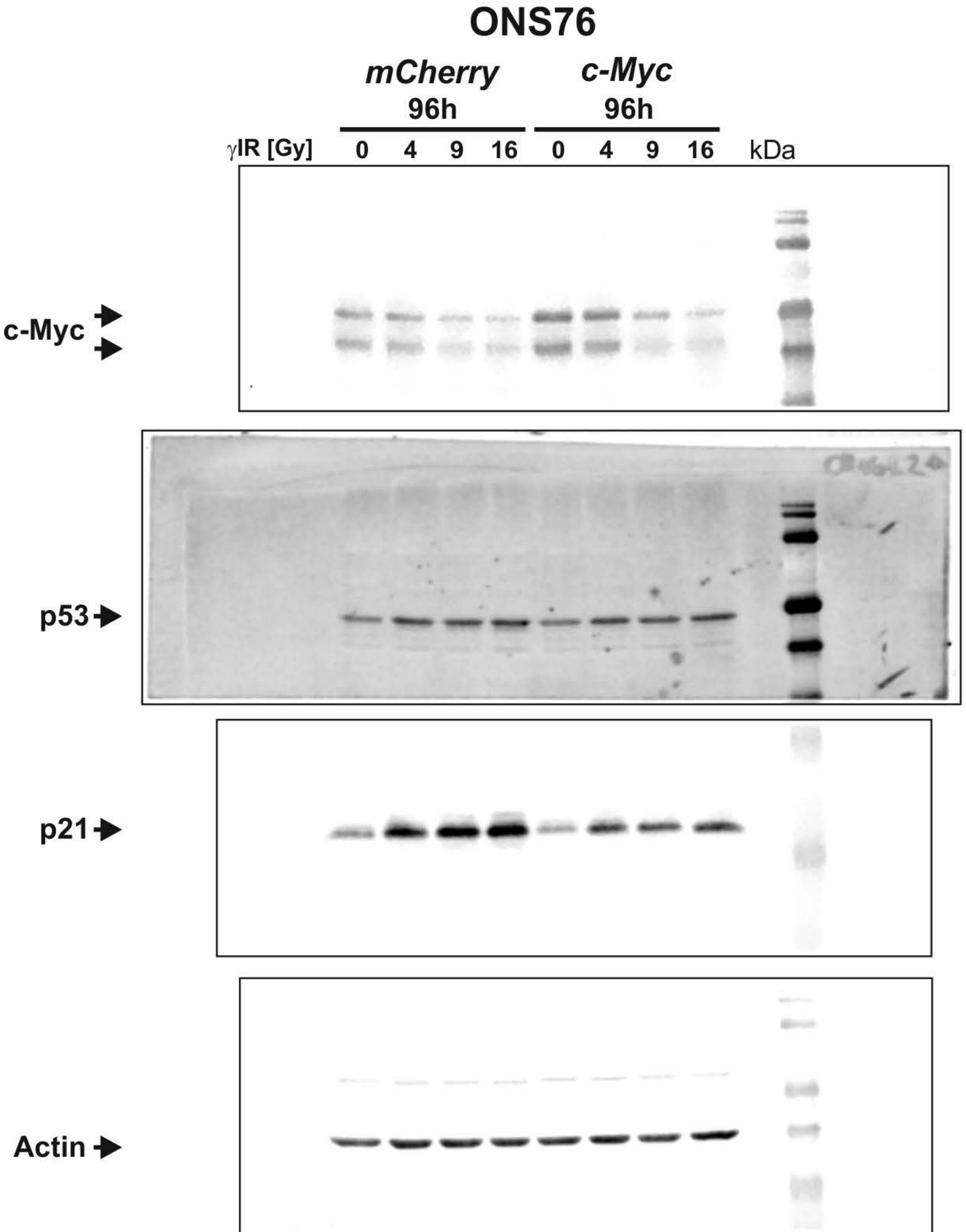
ONS76
mCherry
96h
c-Myc
96h
γIR [Gy]
0
4
9
16
0
4
9
16
kDa
c-Myc
p53
p21
Actin

Figure 4C

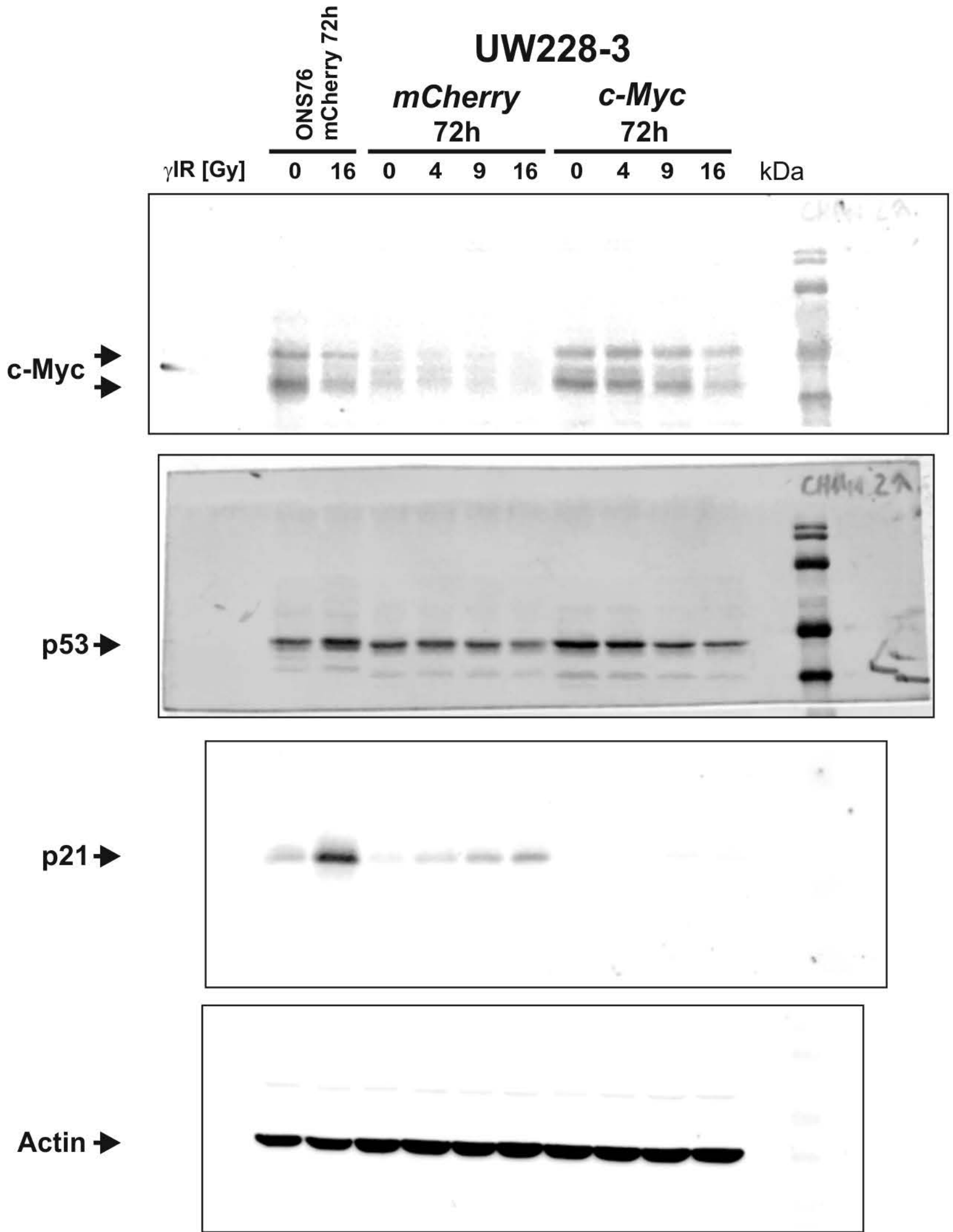
UW228-3
ONS76 mCherry 72h
mCherry 72h
c-Myc 72h
γIR [Gy]
0 16 0 4 9 16 0 4 9 16
kDa
c-Myc
p53
p21
Actin
